# Robust cholesteric liquid crystal elastomer fibres for mechanochromic textiles

**DOI:** 10.1038/s41563-022-01355-6

**Published:** 2022-09-29

**Authors:** Yong Geng, Rijeesh Kizhakidathazhath, Jan P. F. Lagerwall

**Affiliations:** Department of Physics and Materials Science, University of Luxembourg, Luxembourg, Luxembourg

**Keywords:** Polymers, Liquid crystals, Sensors and biosensors

## Abstract

Mechanically responsive textiles have transformative potential in many areas from fashion to healthcare. Cholesteric liquid crystal elastomers have strong mechanochromic responses that offer attractive opportunities for such applications. Nonetheless, making liquid crystalline elastomer fibres suitable for textiles is challenging since the Plateau–Rayleigh instability tends to break up precursor solutions into droplets. Here, we report a simple approach that balances the viscoelastic properties of the precursor solution to avoid this outcome and achieve long and mechanically robust cholesteric liquid crystal elastomer filaments. These filaments have fast, progressive and reversible mechanochromic responses, from red to blue (wavelength shift of 155 nm), when stretched up to 200%. Moreover, the fibres can be sewed into garments and withstand repeated stretching and regular machine washing. This approach and resulting fibres may be useful for applications in wearable technology and other areas benefiting from autonomous strain sensing or detection of critically strong deformations.

## Abstract (web summary)

Robust cholesteric liquid crystal elastomer fibres with rapid and reversible mechanochromic responses are woven and sewn into garments to create smart clothing.

## Main

Smart textiles, capable of sensing and responding to stimuli from the environment^1,2^, are gaining increasing attention across fields as diverse as health care^2,3^, sports^4^ and fashion^5^, motivated by opportunities in, for example, wearable technology^6–9^ and robotics^6,10^, biosensing^11,12^, data collection^13,14^ and information processing^11,12,15^. The actual textile often remains passive, acting as a carrier for electronics that provide functionality but also require a complex device architecture and a power supply, sometimes inhibiting wearing comfort and washability. If the textile fibres themselves are made from a durable responsive material, doors open for fully autonomous smart textiles.

The advancing class of mechanochromic polymers^16^, which change their visual appearance in response to mechanical deformation, can be very powerful in this context if the right type is used. Molecular-scale processes may give too low contrast (Raisch et al. recently reported a wavelength shift of Δ*λ* = 12 nm (ref. ^17^)), and among structurally coloured materials^18,19^, many rely on the incorporation of nanoparticles. It is unclear if such composites resist regular washing procedures, and the nanoparticle incompressibility limits response range and stretchability, which may not reach the requirements for comfortable wearable devices^20^. Moreover, the colour response may be less clear-cut, since the cubic symmetry typical of colloidal crystals yields selective reflection along three orthogonal principal directions: a positive uniaxial strain along one direction produces a positive mechanochromic shift Δ*λ* in this direction but a negative shift in the perpendicular plane. Using a crystal of silica nanoparticles, Cheng et al. obtained Δ*λ* ≈ 50 nm, from weakly red to green, before the material broke at 60% strain^21^. Kim et al. made particle-loaded fibres that elongated 200% before breaking, but the red to green colour shift (Δ*λ* ≈ 0.1 μm) stopped at 80% elongation^22^. Kolle et al. made one-dimensionally (1D) periodic lamellar fibres by rolling an elastomer bilayer around a glass rod^23^, but second-order reflections led to colour mixing, and mechanical durability and washability were not assessed, nor was scalability. The removal of the sacrificial glass rod without damaging the elastomers is a non-trivial challenge for fibres that should be long enough to make garments.

A shining star among mechanochromics is the class of cholesteric liquid crystal elastomers (CLCEs)^24–28^, the self-assembled 1D-periodic helical structure of which gives a fully predictable and reversible mechanochromic response that can span the entire visible spectrum, over a broad elastic range. Using two-dimensional (2D) sheets of CLCE, we recently demonstrated a Δ*λ* = 145 nm shift from red to blue upon 120% strain^29^, and several other groups have reported a similarly impressive CLCE performance^30–40^. However, there have been no reports so far of mechanochromic CLCE fibres, to the best of our knowledge. This is because CLCE helix formation is slow, often not reaching the required uniform alignment^41^ before the Plateau–Rayleigh instability breaks up a one-dimensional fibre of the liquid precursor from which CLCEs are made^42,43^. Non-polymeric cholesterics have been supported on regular fibres^44^ or encapsulated within rubber sheaths^45^, but the liquid state of the cholesteric restrains the mechanochromic response and durability.

Here, we realize CLCE fibres from an oligomeric precursor slightly diluted with solvent, balancing the viscoelastic properties to allow continuous filament extraction, yet delay the Plateau–Rayleigh instability until the helix has developed. We demonstrate a continuous and repeatable mechanochromic response up to Δ*λ* = 155 nm at 200% elongation, and we weave the fibres and sew them into regular fabrics to reveal complex strain patterns, with the fabrics surviving not only long-term repeated use but even several rounds of conventional machine washing.

## Oligomer synthesis and CLCE fibre production

**Fig. 1:**
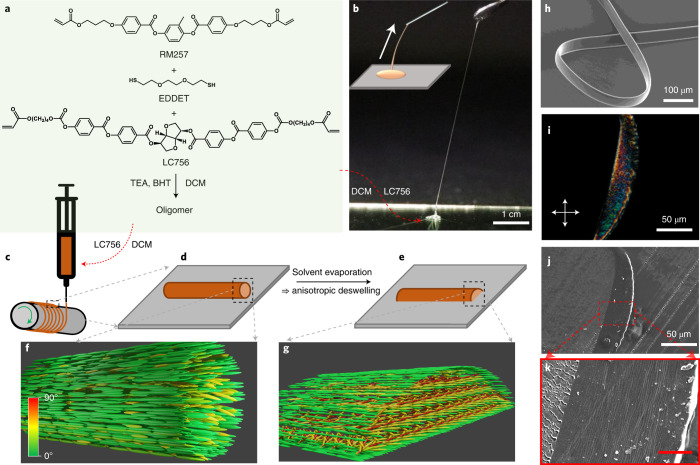
CLCE fibre production from oligomeric precursor liquid. **a**, Synthesis process of the oligomer. **b**, Filaments can be drawn by pulling a needle out of an oligomer solution droplet (schematically illustrated in inset). The red arrow connects the droplet to its components listed in (**a**). **c**–**e**, Schematics (not to scale) illustrating the filament extraction from a syringe containing the oligomer solutioon (as indicated by the red arrow) onto the rotating mandrel (**c**) and the filament shape immediately after deposition (**d**) and after anisotropic deswelling (**e**). **f**,**g**, Idealized schematics (not to scale) illustrating the flow-aligned paranematic state at stage **d** and the ideal cholesteric structure with vertical helix promoted by anisotropic deswelling at stage **e**, respectively. The grey arrows relate identical features shown with different views in different panels, and the colour bar in the lower left corner of **f** indicates the director orientation with respect to the long axis of the filament. **h**, SEM image of CLCE fibre obtained by polymerizing the filament of the precursor liquid. **i**–**k**, Crossed-polarizer microscopy (**i**) and SEM images (**j** and **k**) of a 5-μm-thick cross-sectional slice of the fibre embedded in NOA glue (isotropic). Scale bar in **k** is 10 μm. BHT, butylated hydroxy toluene; TEA, triethylamine.

**Extended Data Fig. 1:**
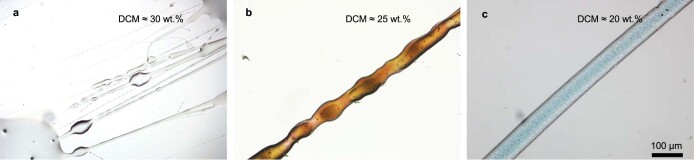
Filaments from CLCE precursor mixtures with different DCM concentrations. **a**, A filament based on LCO1 with 30 wt.% DCM breaks into droplets due to the Plateau-Rayleigh instability. **b**, With 25 wt.% DCM, the filament does not break up but it becomes strongly beaded. **c**, The filament can keep uniform shape with a DCM concentration of 20 wt.%.

The CLCE precursor is based on an acrylate-terminated liquid crystalline oligomer (LCO), synthesized following a thiol–acrylate Michael addition reaction as shown in Fig. 1a (details in Methods). Triethylamine is used as a catalyst to initiate the click reaction between a liquid-crystal-forming diacrylate monomer (RM257, described in the Methods), a polymerizable chiral dopant (LC756, described in the Methods) and a dithiol chain extender (2,2-(ethylenedioxy) diethanethiol, EDDET). To ensure acrylate termination, the reaction is performed with excess (3 mol%) of acrylate monomer. By varying the catalyst concentration, we prepare LCOs of three different lengths (Methods for details). Unless otherwise stated, fibres are made with the shortest oligomer, LCO1, yielding the highest crosslink density since the end groups are reactive. The LCO is diluted with dichloromethane (DCM) at 20% (for LCO1) by mass to form the precursor. The solvent concentration is key to allowing stable filament formation: too little DCM makes the precursor too hard, such that it fractures upon extension into filament shape, while too much DCM renders it too fluid, allowing the Plateau–Rayleigh instability to deform the filament and even break it into droplets before the helix formation is complete (Extended Data Fig. 1).

The central CLCE reflection wavelength *λ*_r_ is defined by the Bragg equation as^41^

1 $${\lambda }_{{\mathrm{r}}}=\bar{n}p\cos \theta ={\lambda }_{0}\cos \theta ,$$

where $\bar{n}\approx 1.6$ is the average refractive index, *p* is the periodicity (pitch) of the helix, *θ* is the angle of incidence with respect to the helix axis and *λ*_0_ is the maximum *λ*_r_ corresponding to *θ* = 0, for illumination and observation along the helix axis (retroreflection). To tune the mechanochromic response regime, we reduce *p* further by dissolving an additional small amount of LC756 into the LCO–DCM precursor mixture. Adding 1.5% or 3.15% (by mass) of LC756, we obtain CLCEs with *λ*_0_ in the red or green region, respectively, for a relaxed fibre. Photoinitiator is also added at this stage to enable photopolymerization and crosslinking into the CLCE after the helix development is complete.

As shown in Fig. 1b and Supplementary Video 1, it is easy to manually pull long filaments from the precursor. In order to reproducibly make fibres of much greater length and well-controlled dimensions and mechanochromic properties, a simple homemade set-up is designed as schematically shown in Fig. 1c. A seed filament is extracted from a syringe that delivers CLCE precursor at a set feed rate *Q*, and it is attached to one end of a rotating mandrel coated with polyvinyl alcohol (PVA). By translating the syringe along the mandrel at a speed *v* adjusted to the rotation speed *ω*, a continuous filament is wound onto the mandrel (Supplementary Video 2). The filament diameter can be adjusted from micrometres to millimetres by tuning *Q*, *v* and *ω*. The extraction from the syringe into air is expected to give the filament an initially cylindrical shape with circular cross-section (schematic drawings in Fig. 1d,f), but as the precursor wets the mandrel, it deforms within minutes into a hemicylindrical shape.

**Extended Data Fig. 2:**
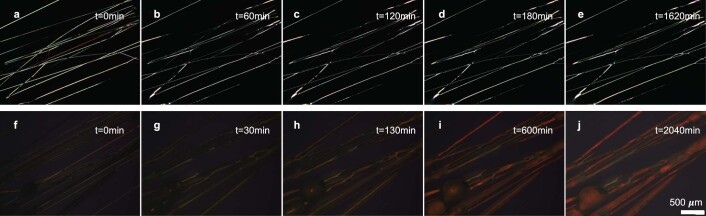
Solvent evaporation and relaxation process. **(a-e)**, Crossed polariser transmission microscopy images of CLCE precursor filaments (oriented parallel with neither polariser) as a function of time after production, showing bright appearance throughout the DCM evaporation process. This proves that long-range order is present from the moment the filament is extruded, initially in the form of a flow-induced paranematic state. **(f-j)**, Crossed polariser reflection microscopy images of filaments (not the same as in a-e) as a function of time after production, showing red selective retroreflection starting to build up after around 2 hours, reaching its near final state around 10 hours after production, not changing substantially after another 24 hours.

Although the precursor at rest is isotropic, the extensional flow during extraction aligns the oligomer uniaxially, yielding a paranematic state with director **n** (direction of long-range orientational order) along the filament (schematically illustrated in a highly idealized fashion in Fig. 1f), as evidenced by the strong birefringence with the uniform optic axis along the filament immediately after extraction (Extended Data Fig. 2, upper row). As the solvent evaporates, the optical characteristics change, and we see the first strong evidence of coloured selective reflection after about 10 hours (lower row). This reveals that the LCO transitions from the flow-aligned paranematic state to the thermodynamically stable cholesteric state, in which **n** continuously twists into a helical arrangement. The filament is compressed unidirectionally during solvent evaporation as the wetting to the mandrel prevents shrinkage in the plane^29^. This ‘anisotropic deswelling’^46^ flattens the filament into a more belt-like shape (Fig. 1h), a process that promotes helix alignment perpendicular to the belt plane^47^. After relaxation, the filament is photopolymerized into the final CLCE fibre by ultraviolet (UV) irradiation. The ideal-case structure promoted by anisotropic deswelling is illustrated schematically in Fig. 1g. The overall experimental evidence to be presented below suggests that this is a suitable approximate model for analysing the fibre behaviour, but there are non-negligible local variations.

To assess the helix alignment uniformity, we slice a thin fibre embedded in UV-cured glue (Norland Optical Adhesive, NOA) using a microtome, studying it using polarizing optical microscopy (POM; Fig. 1i) as well as scanning electron microscopy (SEM; Fig. 1j,k). The colour of the slice in POM is uniform over large areas; it shows nearly no birefringence effect when the belt plane is parallel or normal to the polarizer, and with a first-order *λ* plate inserted, the total birefringence is reduced/increased when the belt normal is along/perpendicular to the slow axis of the *λ* plate (Supplementary Video 3). This behaviour confirms a helix with submicrometre pitch oriented predominantly perpendicular to the belt plane. In SEM, a periodic set of lines largely parallel to the belt plane are visible (Fig. 1k), corroborating the helix orientation. In the Supplementary Discussion, we consider what may be the origin of these lines, and in Supplementary Note 1, we review the effect of increasing the fibre thickness: while the average helix orientation remains perpendicular to the belt plane for thick fibres also, the quality of alignment decreases.

## Selective reflection and mechanochromic response

**Fig. 2:**
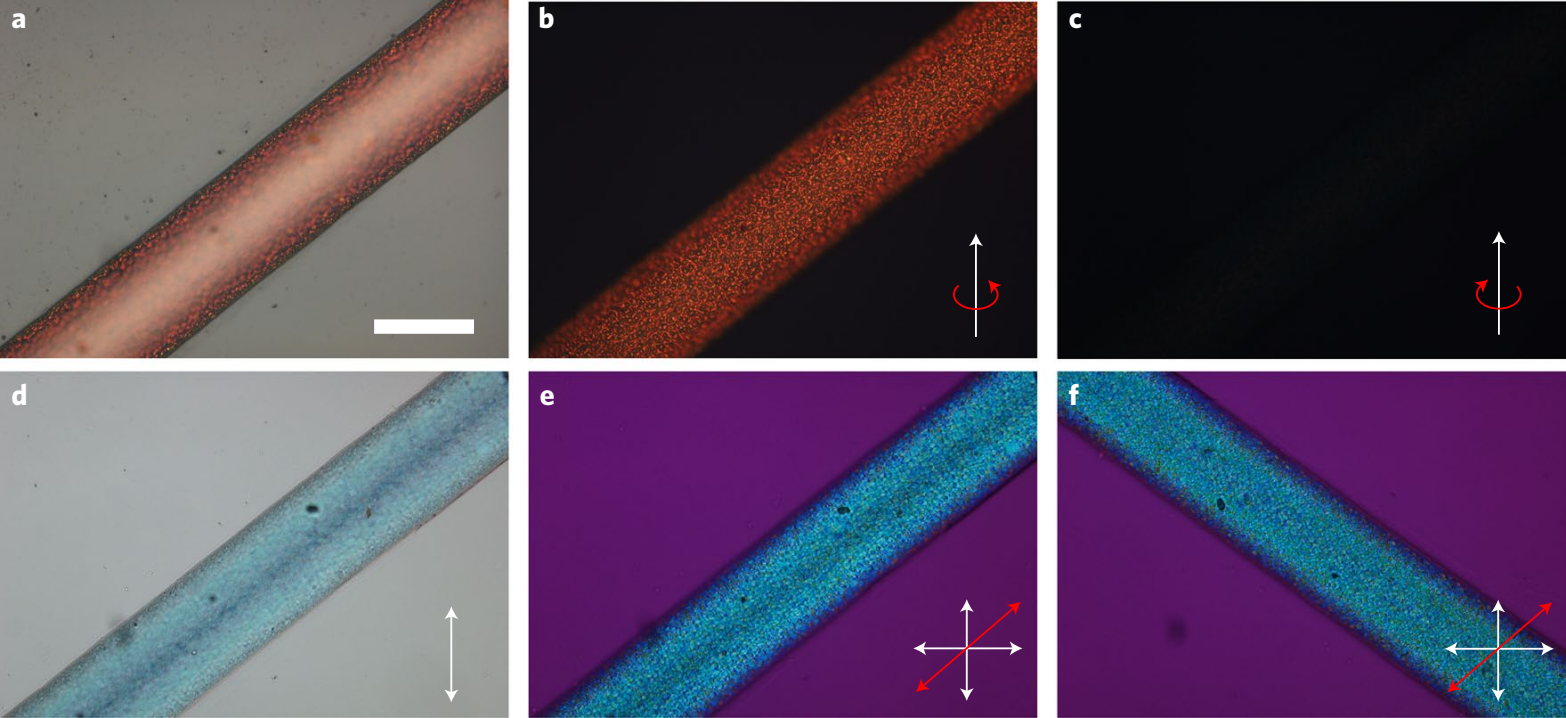
Microscopic CLCE fibre characterization. **a**–**c**, Reflection microscopy images of CLCE fibre without polarizer (**a**), and with right-handed (**b**) and left-handed (**c**) polarizers (scale bar in **a** is 200 μm), revealing behaviour congruent with a right-handed helix oriented perpendicular to the fibre belt plane. **d**–**f**, Transmission microscopy images without analyser (**d**) and between crossed polarizers with a wave-plate inserted with the slow axis along (**e**) and perpendicular to (**f**) the fibre. The blue colour is the complementary colour when red is selectively reflected back to the light source, and the lack of impact of the phase plate corroborates a predominantly vertical helix orientation.

**Extended Data Fig. 3:**
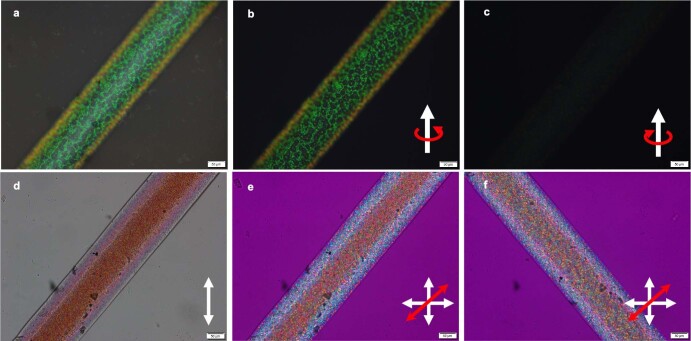
Microscopic characterization of initially green-retroreflecting CLCE fibre. **(a-c)**, Reflection microscopy images of CLCE fibre without polariser, and with right- and left-handed polarisers, respectively (scale bar is 50 *μ*m). The images are fully compatible with a right-handed helix quite well-aligned perpendicular to the fibre belt plane, although the weak remaining reflection for left-handed polarisation reveals that the alignment is slightly worse than for the red *λ*_0_ fibre studied in Fig. 3 in the main paper, and/or that the helix is in a slightly distorted state, changing the ellipticity of the reflected light polarisation slightly away from circular. **(d-f)**, Transmission microscopy images without analyser (d) and between crossed polarisers with waveplate inserted with slow axis along (e) and perpendicular to (f) the fibre. The red colour is the complementary when green is reflected back to the light source. Some change for fibre along and perpendicular to the wave plate slow axis shows that the fibre exhibits weak birefringence, again attributable to slight misalignment or distortion of the helix.

We also carry out detailed POM investigations of the intact CLCE fibres: an ~250-μm-wide fibre with red *λ*_0_ shown in Fig. 2 and one with green *λ*_0_ in Extended Data Fig. 3. The fibres are uniform in width and thickness and show intense selective reflection, clearly visible even with a bright background (Fig. 2a). The Bragg reflection from a well-aligned CLCE should be circularly polarized with the same handedness as the helix^41^. To test this, we insert a *λ*/4 plate in the POM instrument, finding the red fibre colour enhanced with the polarizers set for right-handed circular polarization (Fig. 2b), while with left-handed polarization (Fig. 2c), the fibre is nearly invisible. In transmission without analyser (Fig. 2d), the fibre appears with the complementary blue colour (reflected red light subtracted). Between crossed polarizers with a *λ* plate inserted, the blue colour remains, almost unaffected by fibre rotation along (Fig. 2e) or perpendicular to (Fig. 2f) the slow axis of the *λ* plate (Supplementary Video 3), confirming negligible apparent birefringence from the final fibre viewed along this direction. The overall behaviour is congruent with a vertically aligned right-handed helix with submicrometre pitch^41^, but the top surface shows a speckle pattern with a characteristic size on the order of ~10 μm, suggesting irregularities and some variability in helix orientation near the CLCE–air boundary. Here, the precursor most likely benefited less from anisotropic deswelling due to rapid evaporation of the solvent with consequent rapid viscosity increase; the latter is a well-known effect leading to ‘skin formation’ in polymer fibre spinning^48^. Without the solvent, the cholesteric LCO may also have its lowest surface energy at an interface to air for normal **n** at the boundary, in conflict with the initial flow alignment and the effect of anisotropic deswelling, thus causing local frustration. In the fibre with green *λ*_0_ (Extended Data Fig. 3), made with a higher concentration of LC756, the surface irregularities are slightly more pronounced, although the fibre appears clearly green coloured to the naked eye.

**Fig. 3:**
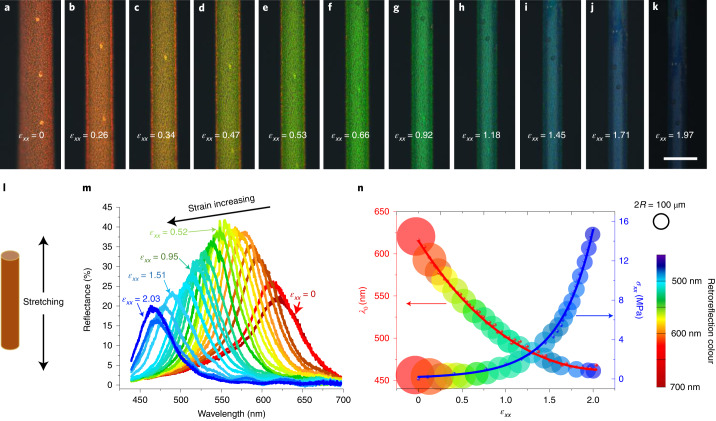
Mechanochromic response of the CLCE fibres. **a**–**k**, Reflection-mode POM images of an initially red-retroreflecting CLCE fibre under elongational strain (scale bar in **k** is 200 μm). **l**, Schematic showing the stretching of the fibre. **m**, Spectra of the selectively reflected light (obtained through a right-handed circular polarizer) corresponding to **a**–**k**. The *y* axis shows the reflectance with respect to unpolarized light. **n**, Central retroreflection wavelength *λ*_0_ and stress *σ*_*xx*_ versus engineering strain *ϵ*_*xx*_ (error bars represent the random observational errors of the instruments used). The red line is a best fit of Warner–Terentjev theory to the *λ*_0_(*ϵ*_*xx*_) data, whereas the blue line is a guide to the eye, obtained as ${\sigma }_{xx}={{\mathrm{e}}}^{-1.82}+1.84{\epsilon }_{xx}+0.22{\epsilon }_{xx}^{2}$. Symbol size represents fibre width (scale indicated by ring of radius *R* at top right).

**Extended Data Fig. 4:**
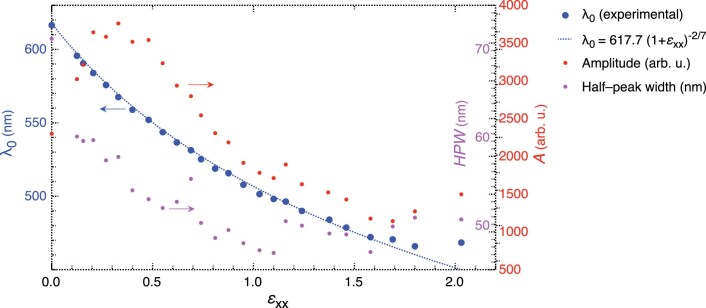
Quantitative comparison of experimental mechanochromic response and theoretical prediction. Central retroreflection wavelength *λ*_0_, half-peak width (*HPW*) and peak area (*A*) (as obtained by fitting a single-peak Lorentz function to the reflection spectra shown in Fig. 3m in the main paper) for an initially red-retroreflecting fibre as a function of uniaxial engineering strain *ϵ*_*x*__*x*_ along the fibre direction, together with the best fit of the function ${\lambda }_{0}={\lambda }_{0}^{* }/{(1+{\epsilon }_{xx})}^{2/7}$ predicted by Warner and Terentjev.^38,49^ Here ${\lambda }_{0}^{* }$ is the central reflection wavelength in the absence of strain, coinciding with the experimentally obtained *λ*_0_ of the unstrained fibre within 1.2 nm. The peak width decreases somewhat with increasing strain, whereas the area initially increases to a maximum around *ϵ*_*xx*_ ≈ 0.3, after which it decreases monotonically throughout the range of overlap between experimental data and Warner-Terentjev theory. We attribute the initial area increase to an initial improvement of the vertical helix alignment, since the elastic compression perpendicular to the fibre upon uniaxial tensile strain along it has an analogous effect as the compression due to anisotropic deswelling during solvent evaporation from the precursor filament.^47^ Also the reduced *HPW* is reasonable in view of a more uniform helix orientation, as the axis of periodicity is better defined, thus narrowing down the Bragg reflection band. The subsequent decrease in area is expected, as the helical structure becomes increasingly distorted upon increasing strain.

The fibre with red retroreflection in its relaxed state provides an ideal mechanochromic response, as the full visible spectrum is available for revealing the magnitude of the elongational strain to which the fibre is subjected (Fig. 3a–k). As shown in Supplementary Videos 5 and 6, the response is immediate, fully reversible and visible to the naked eye even against a bright background. To quantify the response, we measure the reflectance spectra as well as tensile stress *σ*_*xx*_ as a function of engineering strain ${\epsilon }_{xx}={{\Delta }}{l}_{x}/{l}_{x}^{* }$, where the Cartesian coordinate axis $\hat{x}$ is the fibre’s long axis, ${l}_{x}^{* }$ is the original length and Δ*l*_*x*_ is the extension along the fibre. The results are plotted in Fig. 3m,n. At each strain level, we see a clear peak in the reflection spectrum from which we can extract *λ*_0_ (Fig. 3m). For a monodomain CLCE with a helix along $\hat{z}$ that is uniaxially elongated along $\hat{x}$, theory predicts^38,40,49^ that the $\hat{z}$ compression—and thus the mechanochromic blueshift—follows a power law $1/{(1+{\epsilon }_{xx})}^{2/7}$. We fit this function to our experimental *λ*_0_(*ϵ*_*xx*_) data, i.e., retroreflection wavelength as a function of elongational strain, yielding very good results up to a strain of about 160%, after which the reduction in *λ*_0_ levels off up to 200% strain (Extended Data Fig. 4). In Fig. 3n, we visualize the mechanochromic response by plotting a subset of the experimental data against the left *y* axis, using circular symbols with the colour of reflection at each *ϵ*_*xx*_ value and a radius shrinking like the fibre width. The elongational stress *σ*_*xx*_ is plotted in the same way as a function of *ϵ*_*xx*_ against the right *y* axis.

**Extended Data Fig. 5:**
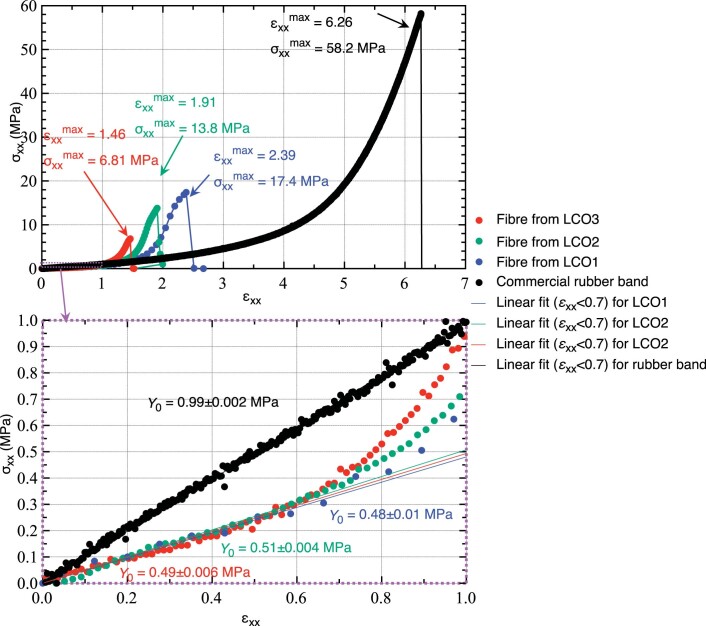
Comparison of mechanical properties of CLCE fibers and a commercial rubber band. Strain-stress curves for pristine fibres made from each oligomer type, upon increasingly strong normal elongational strain *ϵ*_*xx*_ along the fibre axis until the point of rupture, measuring the corresponding normal stress *σ*_*xx*_ at every step. The lower diagram is an inset of the upper (as indicated by the purple frames and arrow) to highlight the linear regime in which the initial modulus was obtained by fitting. The initial modulus *Y*_0_ is independent of oligomer type, whereas the maximum (breaking) strain ${\epsilon }_{xx}^{max}$ and stress ${\sigma }_{xx}^{max}$ increase monotonically from fibres made from LCO3 to fibres made from LCO1. A comparison with a standard (natural rubber) commercial rubber band (data plotted in black) shows that the CLCE fibre strain-stress behavior is qualitatively similar except near the point of rupture, where Young’s modulus starts to level off just before the CLCE fibres break, in contrast to the rubber band modulus which maintains its second-stage strong rise until breaking.

**Extended Data Fig. 6:**
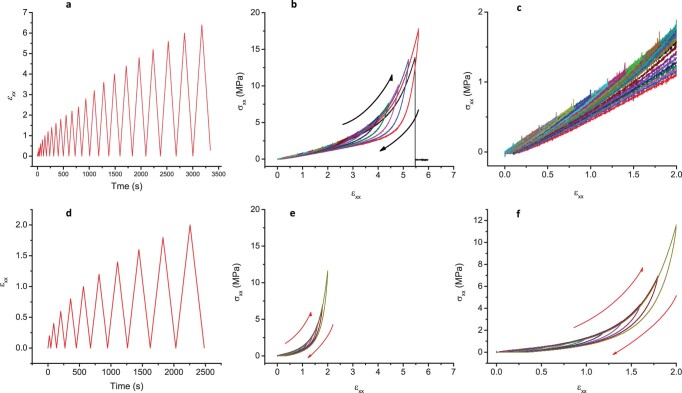
Cycled strain-stress response upon gradually inreasing maximum strain. We subject a commercial rubber band (a-c) and a ground state red-retroreflecting CLCE fibre made from LCO1 (d-f) to repeated strain-stress cycles at gradually increasing maximum strain, the speed being constant throughout the experiment. For the rubber band, we continue up to the point of rupture, whereas the CLCE fibre is strained only up to *ϵ*_*xx*_ = 2. Diagrams a/d show the applied strains *ϵ*_*xx*_ as a function of experiment time whereas b-c/e-f show the hysteresis behavior of *σ*_*xx*_(*ϵ*_*xx*_), with *ϵ*_xx_-axis scaling adapted to the rubber band (b/e) and to the CLCE fibre (c/f), respectively. We find qualitatively similar behavior, although the hysteresis of the rubber band for strains exceeding the initial linear regime actually is larger than that of the CLCE fibre under the corresponding straining conditions. We note that the breaking stress ${\sigma }_{xx}^{max}$ for the rubber band here is only about 1/3 of that in Extended Data Fig. 5, although the breaking strain is comparable (${\epsilon }_{xx}^{max}\approx 5.5$ here compared to 6.3 in Extended Data Fig. 5). This suggests that plastic deformation starts setting in during the cycles at gradually increasing maximum strain, leading to different breaking stress compared to the single increasing straining until break in Extended Data Fig. 5. For the CLCE fibre, the maximum ${\epsilon }_{xx}^{max}=2$ corresponds to ${\sigma }_{xx}^{max}\approx 12$ MPa which is about 20% higher than in Extended Data Fig. 5 for the same strain. We attribute this difference primarily to the slight variability between individual fibres in our non-automated CLCE fibre production.

To assess mechanical durability, we subject fibres to strain–stress cycles in a commercial mechanical testing unit, characterizing them optically before and after the tests. We find (Extended Data Fig. 5) initial Young’s moduli *Y*_0_ = 0.5 MPa for all CLCE fibres regardless of LCO, to be compared to *Y*_0_ = 0.99 MPa for a commercial rubber band. The maximum (max) strain and stress, beyond which the fibres break, differ more, decreasing from LCO1 fibres with ${\epsilon }_{xx}^{{\mathrm{max}}}=2.39$ and ${\sigma }_{xx}^{{\mathrm{max}}}=17.4 \, {\mathrm{MPa}}$ to the least crosslinked fibre made of LCO3 with ${\epsilon }_{xx}^{{\mathrm{max}}}=1.46$ and ${\sigma }_{xx}^{{\mathrm{max}}}=6.81 \, {\mathrm{MPa}}$. The rubber band has ${\epsilon }_{xx}^{{\mathrm{max}}}=6.26$ and ${\sigma }_{xx}^{{\mathrm{max}}}=58.2 \, {\mathrm{MPa}}$. The overall strain–stress behaviours of the CLCE fibres and rubber band are qualitatively similar (Extended Data Fig. 6), but the initial Young’s modulus of the CLCE fibres is about half as high, and the breaking strain and stress are currently substantially lower. Extrapolating the trend of fibres made of the three LCOs, we conjecture that further increase of the crosslink density should increase the strength without losing the mechanochromic response (Supplementary Discussion).

**Extended Data Fig. 7:**
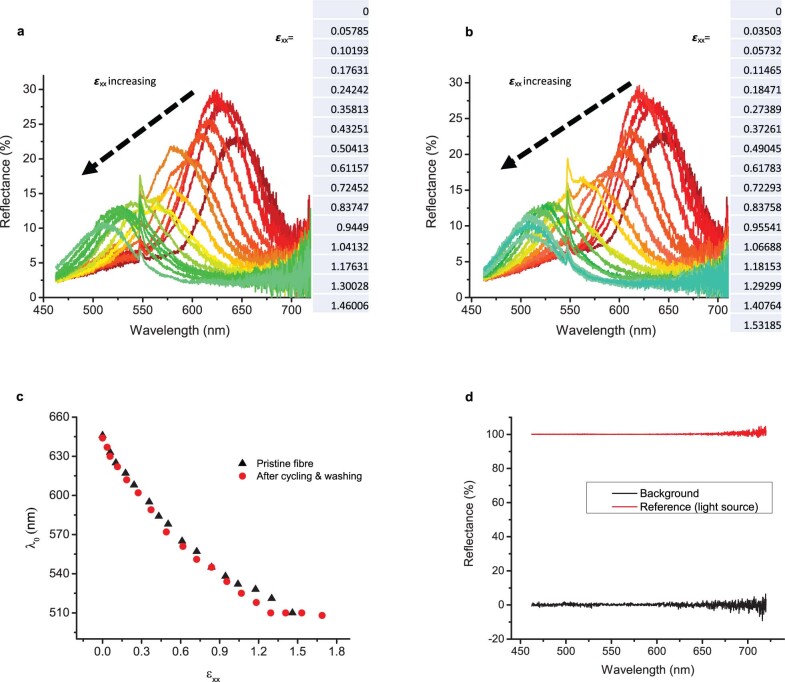
Demonstration of durability of the mechanochromic response of CLCE fibres. The reflection spectra as a function of elongational strain *ϵ*_*xx*_ are measured for a ground state red-retroreflecting CLCE fibre made from LCO1 in its pristine state (a) and then again after it has been subjected to a total of 200 complete strain-stress cycles with *ϵ*_*xx*_ = 0 → 2 → 0 as well as ten complete laundry cycles in a conventional washing machine (b). The central retroreflection wavelength *λ*_0_ at each strain level, as extracted from the spectra, are shown in (c). The behaviour is practically identical before and after the experiment, confirming the excellent durability of the CLCE fibres. To confirm that there are no artifacts in the spectrophotometer measurements, spectra without sample are provided in (d), showing no background signal and 100% reflectance for a reference signal.

**Extended Data Fig. 8:**
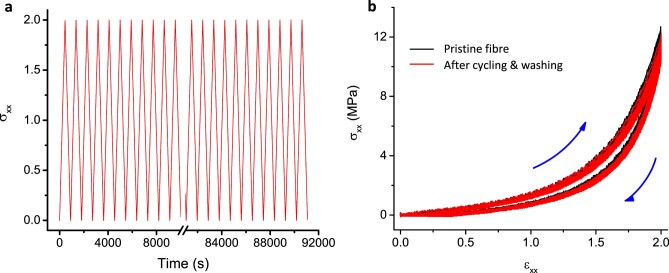
Long-term durability test of CLCE fibre. A ground state red-retroreflecting CLCE fibre made from LCO1 is subjected to 100 complete strain-stress cycles with *ϵ*_*xx*_ = 0 → 2 → 0, followed by ten complete laundry cycles in a conventional washing machine, followed by another 100 cycles *ϵ*_*xx*_ = 0 → 2 → 0. The *σ*_*xx*_(*ϵ*_*xx*_) data for all 200 cycles are plotted in (b), showing no sign of aging or deterioration due to the laundry.

To assess the long-term durability under realistic usage conditions, we measure the reflection spectra of a pristine LCO1-derived fibre for strains up to *ϵ*_*xx*_ = 1.5 (Extended Data Fig. 7a) and then subject it to 100 cycles of *ϵ*_*xx*_ = 0 → 2 → 0. Because an important criterion for smart textiles is washability, we then run the fibre through ten full laundry cycles in a conventional washing machine. After air-drying, we subject the fibre to another 100 cycles of *ϵ*_*xx*_ = 0 → 2 → 0, after which we again measure the mechanochromic response up to *ϵ*_*xx*_ = 1.5 (Extended Data Fig. 7b). There is no change in the strain–stress curves beyond experimental variability (Extended Data Fig. 8), and the mechanochromic response is practically intact, with a *λ*_0_(*ϵ*_*xx*_) relationship that is nearly identical to that of the pristine fibre. These data clearly show that it is realistic to use the fibres in smart textiles; hence, we end the paper with demonstrations of such applications.

## Demonstration of application potential in smart textiles

**Fig. 4:**
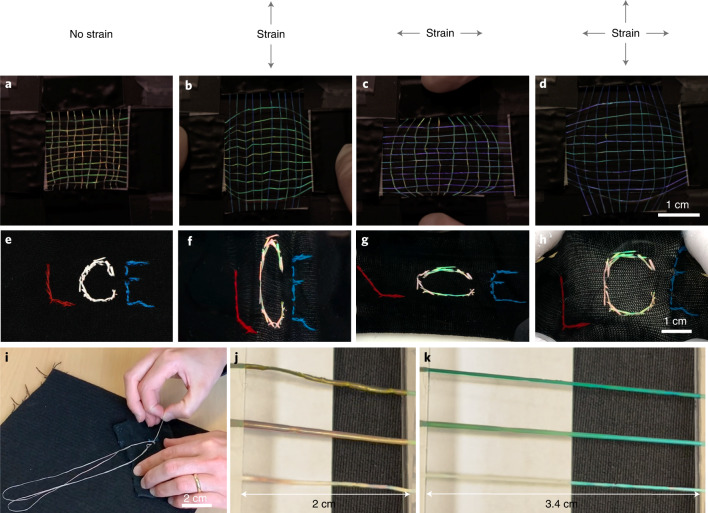
Macroscopic mechanochromic response of CLCE fibres. **a**–**h**, Large-scale view of uniaxial and biaxial mechanochromic response under ambient light of a simple weave of CLCE fibres (**a**–**d**; from Supplementary Video 7), and a single long CLCE fibre sewn into a passive elastic cloth in the shape of a ‘C’ (**e**–**h**). Grey arrows at the top represent the mechanical strain directions. **i**, Snapshot of the sewing procedure from Supplementary Video 8. **j**,**k**, Three sections cut from one CLCE fibre with red ground-state retroreflection, without (bottom), with medium (middle) and with high (top) concentrations of Sudan Black, relaxed (**j**) and stretched (**k**) across a black cloth next to a white paper (from Supplementary Video 9).

We prepare two set-ups for assessing the mechanochromic response as seen on a macroscopic scale by the naked eye under ambient illumination, in a textile context. First, we make a simple weave of ten fibres constituting the warp (vertical in Fig. 4a–d), with each fibre having its ends glued to two movable glass slides at the top and bottom of the photo. Another eleven fibres are woven up and down through the warp as the weft, and their ends are glued to another set of movable glass slides, oriented perpendicular to the first. Fibres with red and green ground-state colour appear in the weave. Supplementary Video 7 shows the weave’s dynamic response to stretching. When stretching the warp (Fig. 4b), its fibres turn green and then blue, or blue and then violet, depending on the ground-state colour. The weft fibres change less, as their deformation is minor in this setting. Upon relaxing the warp, its fibres immediately regain their original colour. If we instead stretch the weft (Fig. 4c), the same behaviour is seen with the roles inverted. When both warp and weft are stretched (Fig. 4d), all fibres are simultaneously blueshifted.

**Extended Data Fig. 9:**
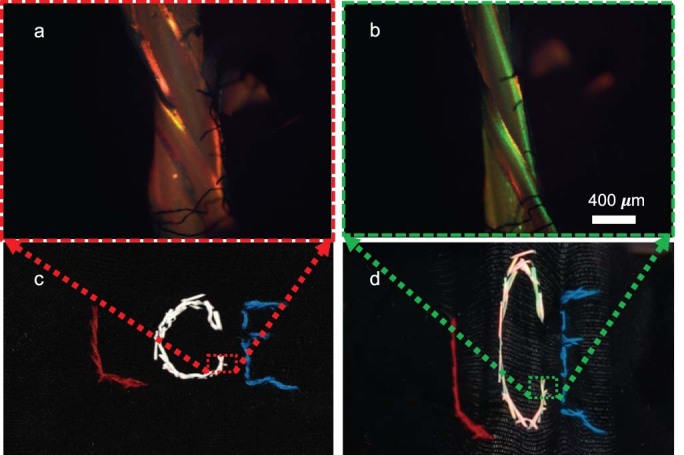
Micro- and macroscopic views of the mechanochromic response of an LCO1-derived initially red reflecting CLCE fibre sewn into an elastic cloth. **(a-b)**, Reflection microscopy images of the CLCE fibre (showing multiple segments overlapping at the intersection between two stitches) in the relaxed state (**a**) and under elongational strain (**b**) at a position indicated in **c** and **d** (repeated from Fig. 4e-f, respectively).

**Extended Data Fig. 10:**
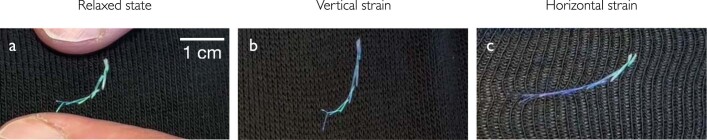
Detecting strain orientation using a CLCE fibre sewn into a cloth along a curve. A CLCE fibre made from LCO1 that is green under ambient light in the relaxed state (a) is sewn into a stretchy black cloth along a continuous curve, such that the stitch changes orientation by 90^∘^ from the beginning to the end. Upon stretching the cloth vertically (b), the vertically sewn stitches turn violet while the horizontal ones retain their original green colour, and the intermediate stitches show a continuous color variation between green and blue depending on the relative angle between stitch and strain direction. For horizontal strain of the cloth (c), the fibre responds in the opposite way, the colour play now ranging from green to violet from vertical to horizontal stitches. The photos are still frames from Supplementary Video 8.

Next, we hand sew (Fig. 4i and Supplementary Video 8) a long (~1 m) CLCE fibre into the shape of the letter ‘C’ in an elastic cloth, surrounded by the letters ‘L’ and ‘E’ sewn using regular yarn. Although the fibre is tuned for red ground-state retroreflection (Extended Data Fig. 9a), it appears with a pink tone under ambient light (Fig. 4e). We attribute this to the less uniform helix orientation in this thicker fibre (Supplementary Note 1), made by reducing the mandrel rotation speed *ω* and translation speed *v*. Because ambient light effectively illuminates the fibre from all directions, domains with different helix orientations then induce Bragg reflections with different angles *θ*, hence varying the blueshifts, explaining the less saturated ground-state colour. The effect is much less pronounced for fibres with green ground-state retroreflection, since the high-*θ* reflections are in the invisible UV region. Indeed, such fibres appear to the naked eye under ambient light as clearly green in the ground state (Extended Data Fig. 10 and Supplementary Video 8.)

As the cloth is stretched in the vertical direction (Fig. 4f), parts of the ‘C’ with vertical fibre orientation blueshift, but the extent depends on the stitch orientation and tautness. The fibre colour reverts immediately and completely when the cloth is relaxed. When it is stretched horizontally (Fig. 4g), sections with taut horizontal stitches turn green. Under biaxial strain (Fig. 4h), mainly these horizontally taut sections turn green; the stretching is done by hand, yielding a biaxial strain with a weaker vertical component compared to the case in Fig. 4f. This illustrates the power of CLCE fibres in revealing complex strain patterns. As a further demonstration, we ‘program’ a fibre to monitor in-plane strains by sewing it along a 90° arc into a cloth (Supplementary Video 8 and Extended Data Fig. 10). For any uniaxial strain, a range of mechanochromic responses is seen along the arc, with each stitch showing a different colour depending on its angle with respect to the strain direction.

Based on our prior success in making 2D CLCE films of several millimetres thickness with a uniform helix by ensuring anisotropic deswelling from both opposing film surfaces^29^, we anticipate that uniform helix orientation can be achieved also in thick fibres if the mandrel design and material are adapted accordingly. A simpler solution, which also boosts the colour contrast against bright backgrounds, is to add a black dye to the fibre, which absorbs undesired light scattering. We do this by soaking fibres in a solution of Sudan Black (Supplementary Methods), resulting in excellent visibility of the mechanochromic response, even under ambient light over a white paper (Fig. 4j,k).

In summary, we have developed a simple procedure for making long CLCE fibres that exhibit an excellent mechanochromic response, spanning the entire visible colour spectrum, upon elongational strain up to 200%. To scale up production, longer mandrels with larger diameter can be used, and by automating the syringe translation along the mandrel, the precursor solution can be deposited with less gap between adjacent turns of the filament. Because of three factors—the fibres can be woven or sewn into regular elastic garments, our experience suggests that this will not impair user comfort, and the fibres survive long-term use as well as repeated machine washing—they can be used as smart textiles that reveal even complex strain patterns. We believe this will be particularly useful in sports clothing and wearable robotics, but it also offers ample opportunities for innovative fashion and artistic applications. Moreover, in non-wearable contexts, the CLCE fibres might serve important functions, for instance, in furniture or as a warning sign in rope (that is, a rope with incorporated CLCE fibres can signal if it is being strained to dangerous levels, or if it has been subject to strains leading to plastic deformation, such that it should be discarded).

## Methods

### Oligomer formulation

The molecular structures of the monomers used to prepare the LCOs from which the CLCE fibres were prepared are shown in Fig. 1a. The diacrylate mesogen RM257 (1,4-bis-(4-(3-acryloyloxypropyloxy)benzoyloxy)-2-methylbenzene; 1.92 g, 1 equiv.; Wilshire Technologies), the dithiol monomer EDDET (0.59 g, 1 equiv.; Sigma-Aldrich), the chiral dopant LC756 ((3*R*,3a*S*,6a*S*)-hexahydrofuro[3,2-b] furan-3,6-diyl bis(4-(4-((4-(acryloyloxy)butoxy) carbonyloxy) benzoyloxy)benzoate); 85 mg, 0.03 equiv.; Synthon Chemicals) and BHT (5.3 mg, 0.2 wt%; Sigma-Aldrich) were dissolved in DCM (6 ml). Then, TEA was added as a catalyst for the first-stage Michael addition reaction, and the solution was stirred at room temperature for 24 h. The LCO length decreases with increasing TEA concentration; hence, we varied its volume from 0.5 ml for LCO3 (number and weight average molar masses *M*_n_ = 12.0 kg mol^–1^, *M*_w_ = 19.5 kg mol^–1^; dispersity index = 1.63) to 1.0 ml for LCO2 (*M*_n_ = 11.5 kg mol^–1^, *M*_w_ = 18.9 kg mol^–1^; dispersity index = 1.64) to 1.6 ml for LCO1 (*M*_n_ = 10.8 kg mol^–1^, *M*_w_ = 17.7 kg mol^–1^; dispersity index = 1.63). The resulting mixture was washed thrice with 1 M aqueous HCl to remove TEA. The organic layer was washed with brine solution, dried over MgSO_4_ and then filtered. The solvent was evaporated using a rotary evaporator to yield the LCO. The LCO masses and dispersity index values were determined by gel permeation chromatography.

To prepare the CLCE precursor solution, the LCO was mixed in DCM with additional LC756 (1.5 to 3.15 wt% for red and green retroreflection, respectively) and a photoinitiator 2,2-dimethoxy-2-phenylacetophenone, Irgacure 651 (Sigma-Aldrich) at a concentration in the range 1–4 wt%. The overall concentration of DCM was 20 wt% for LCO1, 22 wt% for LCO2 and 25 wt% for LCO3. All chemicals were used as received.

### Fibre production

To produce the fibres, we first coated the outside of a 20 ml plastic syringe barrel acting as the mandrel with a thin layer of PVA. This was done manually using a brush dipped into a 15 wt% aqueous solution of PVA (Aldrich, *M*_w_ = 85−124 kg mol^–1^, 87−89% hydrolysed). The solution viscosity was high enough that the coating remained flat during drying, without breaking up due to the Rayleigh–Taylor instability. After drying (minimum 1 hour), the oligomer precursor mixture was extruded from a 10 ml syringe mounted in a Cronus syringe pump through a blunt orifice needle and collected on the mandrel, set in constant slow rotation driven by a stepper motor. The lateral translation of the syringe pump along the length of the mandrel was gently done by hand. The fibre-coated mandrel was placed at room temperature in a fume-hood for 36 hours for annealing the fibres, which were then polymerized using a UV box (Opsytec Dr. Gröbel Irradiation Chamber BSL-01) at 330–450 nm with an intensity of 200 mW cm^–2^ for 5 min. To harvest the fibre, the mandrel was submerged in water for 10–20 minutes, dissolving the PVA layer and allowing the entire fibre length to be easily picked up and suspended in air until dry.

### Characterization of CLCE fibre and its mechanochromic response

An Olympus BX51 polarizing optical microscope equipped with a digital camera (Olympus DP73) was used for microscopic characterization, whereas macroscopic still images and videos were acquired with a Canon EOS 100D digital SLR (single-lens reflex) camera. SEM imaging was done using a JEOL JSM-6010LA, operated in the 5–20 kV range using an in-lens secondary electron detector. Prior to SEM imaging, samples were coated with gold (~3 nm thickness) using a Quorum Q150R ES coater. The reflection spectra were obtained using unpolarized white illumination with an Andor KY328i spectroscopy platform, coupled with an Andor Newton DU920P-BXE2-DD as the detector (Oxford Instruments), connected directly to a microscope. For assessing the polarization of the reflected light, a right- or left-handed circular polarizer was placed in the beam path from the microscope to the spectrophotometer. For practical reasons, a relatively low magnification (×10) objective had to be used; hence, the area *A* over which the spectrum was measured was larger than the area *A*_f_ of the fibre section within the measuring window. Furthermore, *A*_f_ decreased upon increasing fibre elongation, due to the corresponding reduction of the fibre width. To estimate a correct value of the actual fibre reflectance *R*, we assumed that the reflectance value *R*′ given by the spectrophotometer software can be calculated as *R*′ = *RA*_f_/*A*. The normalization was done with a mirror in the sample holder. When neither mirror nor fibre was present, the reflection spectrum was a flat line at *R*′ ≈ 0, apart from noise (Extended Data Fig. 7d). The spectra shown in Fig. 3m were obtained by rescaling the *R*′ value for each data point as *R* = *R*′*A*/*A*_f_, where *A*/*A*_f_ was established by measuring the fraction of the image area of a photo taken at each strain level that was covered by the fibre.

Reflection spectra were plotted using Origin Pro 9.1 (OriginLab), and the central retroreflection wavelength *λ*_0_ was obtained by fitting a single-peak Lorentz function to each reflection peak in the raw data. The Warner–Terentjev *λ*_0_(*ϵ*_*xx*_) function was programmed and fitted to the experimental data using Pro Fit 7 (Quantumsoft). To establish the strain during the *λ*_0_(*ϵ*_*xx*_) experiments, the force *F* required for elongating the fibre was measured using a force gauge (Mark-10, Model M3-5), and the stress was calculated as *σ*_*xx*_ = *F*/*a*, where *a* is the cross-sectional area of the fibre. The latter was established by measuring the ground-state cross-sectional area using a microscope (after fibre rupture) and then calculating the area at each strain level from the elongational strain *ϵ*_*xx*_, assuming volume conservation. For the cycled strain–stress experiments, the fibre under testing was mounted vertically in a Mark-10, F105-IMTE Advanced Test Frame, coupled with FS05-05 Tension and Compression Force Sensors (maximum loads of 2.5 N and 25 N, respectively), fixing its ends using tape. For the ten laundry cycles done after the first 100 cycles of mechanical testing, we placed the fibre in a mesh laundry bag, which we put into a conventional washing machine, running a standard 60 °C programme with detergent and ending with centrifugation. A microtome (Leica RM2200) was used to slice the CLCE fibre with 5 μm steps after it had been embedded in UV-cured NOA glue for support.

### Textile context demonstrations

Weaving and sewing experiments were both done on LCO1-derived fibres. For weaving, fibres were cut into segments of ~3 cm length and placed with about 2 mm separation in both the warp and weft. For the initial sewing experiment, a fibre of about 1 m length was coated with PVA as described in the Supplementary Methods and sewn by hand into a regular black elastic fabric for socks. After sewing was complete, the entire cloth was immersed in water for about 15 minutes to remove the excess PVA.

## Online content

Any methods, additional references, Nature Research reporting summaries, source data, extended data, supplementary information, acknowledgements, peer review information; details of author contributions and competing interests; and statements of data and code availability are available at 10.1038/s41563-022-01355-6.

## Supplementary information

Supplementary Information Supplementary Figs. 1–8, Notes 1–5, Discussion, Methods, video legends and references.

Supplementary Video 1 Manual pulling of the LCO–DCM precursor using a spatula, showing good filament formation ability.

Supplementary Video 2 The fibre production process, with a precursor liquid filament extracted from a syringe and deposited on a rotating mandrel.

Supplementary Video 3 Transmission POM investigations of microtomed cross-sections of LCO1-derived fibres with red ground-state retroreflection colour, of varying thickness, as they are rotated between crossed polarizers.

Supplementary Video 4 Transmission POM investigation of an LCO1-derived fibre with red ground-state retroreflection colour, rotating between crossed polarizers with a first-order *λ* plate (530 nm) inserted.

Supplementary Video 5 Reflection POM imaging of an LCO1-derived fibre with red ground-state retroreflection colour that is being elongated up to 200% strain and then relaxed.

Supplementary Video 6 Macroscopic views with ambient illumination of stretching LCO1-derived fibres with red ground-state reflection colour over black and white backgrounds.

Supplementary Video 7 Macroscopic view of stretching a weave of fibres made from LCO1 in different directions, under ambient illumination.

Supplementary Video 8 Performance of LCO1-derived CLCE fibres during and after hand sewing into elastic fabric: the sewing process and uniaxial and biaxial stretching of two fabrics with CLCE fibres sewn into them.

Supplementary Video 9 Macroscopic views under ambient illumination of uniaxial stretching of LCO1-derived fibres with and without Sudan Black dye, over white and on black backgrounds, and of fibres derived from LCO2 and LCO3.

Supplementary Video 10 Stretching three LCO1-derived fibres with varying chiral dopant concentration, giving them a green, orange or red ground-state reflection colour, as seen macroscopically and in reflection POM.

## Data Availability

All raw data supporting the findings of this study are openly available at https://osf.io/y97sz/?view_only=ecc1f618018646ddbf1cde90a4fb49cf.

## References

[1] Koncar, V. *Smart Textiles and Their Applications* (Woodhead Publishing, 2016).

[2] Van Langenhove, L., Hertleer, C., Westbroek, P. & Priniotakis, J. in *Smart Textiles for Medicine and Healthcare: Materials, Systems and Applications* 106–122 (Woodhead Publishing and CRC Press, 2007).

[3] Hatamie A (2020). Textile based chemical and physical sensors for healthcare monitoring. J. Electrochem. Soc..

[4] Patil VP, Sandt JD, Kolle M, Dunkel J (2020). Topological mechanics of knots and tangles. Science.

[5] Yetisen, A. et al. Nanotechnology in textiles. *ACS Nano* **10**, 3042–3068 (2016). 10.1021/acsnano.5b08176 26918485

[6] Xiong J, Chen J, Lee PS (2021). Functional fibers and fabrics for soft robotics, wearables, and human–robot interface. Adv. Mater..

[7] Ma Z (2021). Permeable superelastic liquid-metal fibre mat enables biocompatible and monolithic stretchable electronics. Nat. Mater..

[8] Veerapandian S (2021). Hydrogen-doped viscoplastic liquid metal microparticles for stretchable printed metal lines. Nat. Mater..

[9] Van Langenhove, L., Puers, R. & Matthys, D. in *Textiles for Protection* Chapter 7, 176–195 (Woodhead Publishing, 2005).

[10] O’Neill, C., McCann, C., Hohimer, C., Bertoldi, K. & Walsh, C. Unfolding textile-based pneumatic actuators for wearable applications. *Soft Robot.* **9**, 163–172 (2022). 10.1089/soro.2020.0064 33481682

[11] Bandodkar AJ, Wang J (2014). Non-invasive wearable electrochemical sensors: a review. Trends Biotechnol..

[12] Wang W (2017). Harnessing the hygroscopic and biofluorescent behaviors of genetically tractable microbial cells to design biohybrid wearables. Sci. Adv..

[13] Son D (2014). Multifunctional wearable devices for diagnosis and therapy of movement disorders. Nat. Nanotechnol..

[14] Guo L, Berglin L, Mattila H (2012). Improvement of electro-mechanical properties of strain sensors made of elastic-conductive hybrid yarns. Text. Res. J..

[15] Park, S., Mackenzie, K. & Jayaraman, S. in *Proceedings of the 39th Annual Design Automation Conference* 170–174 (Association for Computing Machinery, 2002).

[16] Chen Y, Sommer M, Weder C (2021). Mechanochromic polymers. Macromol. Rapid Commun..

[17] Raisch, M., Maftuhin, W., Walter, M. & Sommer, M. A mechanochromic donor-acceptor torsional spring. *Nat. Commun.* **12**, 4243 (2021). 10.1038/s41467-021-24501-1 PMC8270966 34244510

[18] Clough JM, Weder C, Schrettl S (2021). Mechanochromism in structurally colored polymeric materials. Macromol. Rapid Commun..

[19] Chen G, Hong W (2020). Mechanochromism of structural-colored materials. Adv. Opt. Mater..

[20] Bourzac K (2018). Moving skin beyond the biological. Nature.

[21] Cheng C-H (2020). Fabrication and deformation of mechanochromic nanocomposite elastomers based on rubbery and glassy block copolymer-grafted silica nanoparticles. Macromolecules.

[22] Kim JH (2021). Microfluidic production of mechanochromic photonic fibers containing nonclose-packed colloidal arrays. Small Sci..

[23] Kolle M (2013). Bio-inspired band-gap tunable elastic optical multilayer fibers. Adv. Mater..

[24] Warner, M. & Terentjev, E. M. *Liquid Crystal Elastomers* Vol. 120 (Oxford Univ. Press, 2007).

[25] White TJ, Broer DJ (2015). Programmable and adaptive mechanics with liquid crystal polymer networks and elastomers. Nat. Mater..

[26] Van Oosten CL, Bastiaansen CW, Broer DJ (2009). Printed artificial cilia from liquid-crystal network actuators modularly driven by light. Nat. Mater..

[27] Palffy-Muhoray, P. in *Liquid Crystal Elastomers: Materials and Applications* (ed. de Jeu, W.) 95–118 (Springer, Berlin, Heidelberg, 2012).

[28] Kim S-U (2022). Broadband and pixelated camouflage in inflating chiral nematic liquid crystalline elastomers. Nat. Mater..

[29] Kizhakidathazhath R (2020). Facile anisotropic deswelling method for realizing large-area cholesteric liquid crystal elastomers with uniform structural color and broad-range mechanochromic response. Adv. Funct. Mater..

[30] Hisano, K. et al. Mechano-optical sensors fabricated with multilayered liquid crystal elastomers exhibiting tunable deformation recovery. *Adv. Funct. Mater.* **31**, 2104702 (2021).

[31] Zhang P, Zhou G, de Haan LT, Schenning AP (2021). 4D chiral photonic actuators with switchable hyper-reflectivity. Adv. Funct. Mater..

[32] Martinez, A. M., McBride, M. K., White, T. J. & Bowman, C. N. Reconfigurable and spatially programmable chameleon skin-like material utilizing light responsive covalent adaptable cholesteric liquid crystal elastomers. *Adv. Funct. Mater.* **30**, 2003150 (2020).

[33] Zhang P, Shi X, Schenning AP, Zhou G, de Haan LT (2020). A patterned mechanochromic photonic polymer for reversible image reveal. Adv. Mater. Interfaces.

[34] Varanytsia A, Nagai H, Urayama K, Palffy-Muhoray P (2015). Tunable lasing in cholesteric liquid crystal elastomers with accurate measurements of strain. Sci. Rep..

[35] Picot OT (2013). A real time optical strain sensor based on a cholesteric liquid crystal network. RSC Adv..

[36] Serra F, Matranga MA, Ji Y, Terentjev EM (2010). Single-mode laser tuning from cholesteric elastomers using a ‘notch’ band-gap configuration. Opt. Express.

[37] Schmidtke J, Kniesel S, Finkelmann H (2005). Probing the photonic properties of a cholesteric elastomer under biaxial stress. Macromolecules.

[38] Cicuta P, Tajbakhsh A, Terentjev E (2004). Photonic gaps in cholesteric elastomers under deformation. Phys. Rev. E.

[39] Finkelmann H, Kim ST, Munoz A, Palffy-Muhoray P, Taheri B (2001). Tunable mirrorless lasing in cholesteric liquid crystalline elastomers. Adv. Mater..

[40] Mao Y, Terentjev E, Warner M (2001). Cholesteric elastomers: deformable photonic solids. Phys. Rev. E.

[41] Kitzerow, H.-S. & Bahr, C. (eds) *Chirality in Liquid Crystals* (Springer, 2000).

[42] Eggers J (1997). Nonlinear dynamics and breakup of free-surface flows. Rev. Mod. Phys..

[43] Urbanski M (2017). Liquid crystals in micron-scale droplets, shells and fibers. J. Phys. Condens. Matter.

[44] Guan Y, Agra-Kooijman DM, Fu S, Jákli A, West JL (2019). Responsive liquid-crystal-clad fibers for advanced textiles and wearable sensors. Adv. Mater..

[45] Honaker LW, Vats S, Anyfantakis M, Lagerwall JP (2019). Elastic sheath–liquid crystal core fibres achieved by microfluidic wet spinning. J. Mater. Chem. C.

[46] Kim ST, Finkelmann H (2001). Cholesteric liquid single-crystal elastomers (LSCE) obtained by the anisotropic deswelling method. Macromol. Rapid Commun..

[47] Frka-Petesic, B., Kamita, G., Guidetti, G. & Vignolini, S. Angular optical response of cellulose nanocrystal films explained by the distortion of the arrested suspension upon drying. *Phys. Rev. Mater*. **3**, 045601 (2019). 10.1103/PhysRevMaterials.3.045601 PMC7116400 33225202

[48] Guenthner AJ (2006). Dynamics of hollow nanofiber formation during solidification subjected to solvent evaporation. Macromol. Theory Simul..

[49] Warner M, Terentjev EM, Meyer RB, Mao Y (2000). Untwisting of a cholesteric elastomer by a mechanical field. Phys. Rev. Lett..

