# Supplementary material for: Robust cholesteric liquid crystal elastomer fibres for mechanochromic textiles
Source: Nat Mater. 2022 Sep 29;21(12):1441–7. doi: 10.1038/s41563-022-01355-6 (PMC9712110; doi:10.1038/s41563-022-01355-6)
Supplement: Supplementary file 1 — Supplementary Figs. 1–8, Notes 1–5, Discussion, Methods, video legends and references. [file 41563_2022_1355_MOESM1_ESM.pdf]

nature portfolio

https://doi.org/10.1038/s41563-022-01355-6

Supplementary information

Robust cholesteric liquid crystal elastomer fibres for mechanochromic textiles

In the format provided by the authors and unedited

Supplementary Text

Supplementary videos legends

1. Manual pulling of the LCO–DCM precursor using a spatula, which shows good filament formation ability. Video is in real time.

2. The fibre production process, with a precursor liquid filament extracted from a syringe and deposited on a rotating mandrel. Video is in real time.

3. Transmission POM investigations of microtomed cross sections of LCO1-derived fibres with red ground state retroreflection colour, rotating between crossed polarisers; (a) thin fibre, also (b) with a λ plate (530 nm) inserted; (c) medium thick fibre; (d) thick fibre. All videos are in real time.

4. Transmission POM investigation of an LCO1-derived fibre with red ground state retroreflection colour, rotating between crossed polarisers with first-order λ plate (530 nm) inserted. Video is in real time.

5. Reflection POM imaging of an LCO1-derived fibre with red ground state retroreflection colour that is being elongated up to 200% strain and then relaxed. Reflection spectra are obtained in an identical experiment with the spectrophotometer taking the place of the camera. Video is in real time.

6. Macroscopic views with ambient illumination of stretching LCO1-derived fibres with red ground state reflection colour, (a) over black background and (b) over white background. Videos are sped up two times faster than reality.

7. Macroscopic view of stretching of weave of fibres made from LCO1 in different directions, under ambient illumination. Video is in real time.

8. Performance of LCO1-derived CLCE fibres during and after hand sewing into elastic fabric. (a) Hand sewing of a long looped fibre with red ground state retroreflection colour into a fabric in the form of the letter 'C' (video speed twice of reality); (b) Macroscopic view under ambient illumination of uni- and biaxial stretching (by hand) of the fabric with CLCE fibre immersed in water (video speed twice of reality); (c) Macroscopic view under ambient illumination of uniaxial stretching (by hand) in different directions of an elastic fabric into which a shorter fibre with green ground state retroreflection colour has been hand sewn into an arc shape.

9. Macroscopic views under ambient illumination of uniaxial stretching of (a) three segments of an LCO1-derived fibre with red ground state reflection, pristine (bottom) and doped with medium (middle) and high (top) concentrations of Sudan Black, over white and black backgrounds, and (b) an LCO2- (top) and an LCO3-derived (bottom) fibre with orange ground state reflection colour, on black background. Videos are in real time.

10. Stretching three LCO1-derived fibres with varying chiral dopant concentration, giving them green, orange and red ground state reflection colour, respectively, as seen (a) macroscopically and (b) in reflection POM. Videos are real time.

Supplementary Methods

The authors affirm that the human research participant appearing in Supplementary Video 8 (the senior author of the paper) provided informed consent for publication of this video.

Preparation of fibre cross sections for microscopy investigation

To better investigate the helix development as a function of fibre thickness, we embed several fibres in a polymer matrix and slice them perpendicular to the fibre axis using a microtome. The preparation procedure is illustrated schematically in Supplementary Fig. 1. One end of a fibre segment of around 2 cm length is glued to a glass slide, and the other end is attached to a rod placed above the slide such that the fibre segment is maintained perpendicular to the slide. The end on the substrate is now embedded with sufficient amount of UV-curable glue (Norland Optical Adhesive, NOA) to form the surrounding matrix, after which the NOA glue is turned solid by UV irradiation. We now cut the fibre segment from the rod and place the glass slide with NOA-embedded fibre on the microtome sample clamp with the cutting plane parallel to the glass slide. The cutting step size is 5 μm, hence each slice produced is 5 μm thick. The obtained slices are first investigated thoroughly in POM, followed by gold coating in order to allow high-resolution imaging of the surface by SEM.

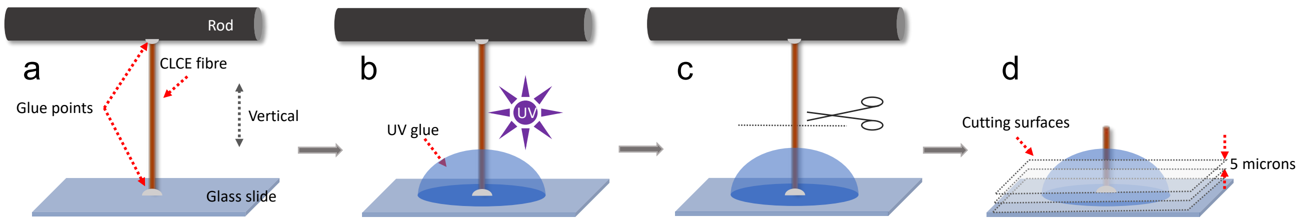

Supplementary Fig. 1. Microtoming fibre for revealing its cross section. A CLCE fibre segment is glued between a glass slide and a rod kept above it (a), surrounded by NOA glue which is UV-cured into a solid (b), after which the excess fibre is cut off (c). The slide is placed on the sample clamp of a microtome which is then used to cut 5 μm thick slices of the fibre (d) with surrounding cured NOA matrix parallel to the slide, hence perpendicular to the fibre axis.

Endurance tests

For the experiment described in Extended Data Fig. 7, we first put the fibre through 100 cycles $\varepsilon_{xx} = 0 \rightarrow 2 \rightarrow 0$ at the same speed as used in Extended Data Fig. 6, measuring σ_{xx} continuously. This is followed by placing the fibre in a conventional consumer washing machine, enclosed in a mesh laundry bag in order that it can easily be retrieved after the experiment, and running ten full laundry cycles at 60°C with detergent, with centrifugation at the end of each cycle. After the tenth laundry cycle, the fibre is dried in air and then we subject it to another 100 cycles $\varepsilon_{xx} = 0 \rightarrow 2 \rightarrow 0$.

Infusing CLCE fibres with black dye

For introducing black dye into CLCE fibres and thus reduce unwanted scattering, the fibre is allowed to swell for 5 minutes in 25 mL of a solution of Sudan Black in a 1:1 (by volume) mixture of dichloromethane and ethanol. The former solvent strongly swells the fibre, hence the latter solvent is added to adjust the swelling pressure, to avoid that the CLCE breaks in the process. The medium and high concentrations of dye in Fig. 4j–k in the main paper were achieved by using 0.07 wt.% and 0.13 wt% of Sudan Black, respectively. The film absorbs the dye while swelling, and afterwards the fibre is dipped into pure ethanol to deswell it, and to wash away excess Sudan Black from the fibre surface.

Hand sewing of CLCE fibres

To minimise the inconvenience of different parts of the same long CLCE fibre sticking together during the hand sewing experiment, we immersed the fibre to be sewn in an aqueous PVA solution while it was still on the mandrel. The fibre was slowly pulled out and left hang-drying. The PVA-coated CLCE fibre was then inserted through the eye of a standard hand sewing needle, after which its ends were tied into a knot to form a single large loop. After sewing was complete, the PVA was easily removed by immersing the entire cloth in a water bath for about fifteen minutes.

Supplementary Notes

Supplementary Note 1: Investigation of cross sections of fibres of varying thickness

Optical and electron microscopy images/movies of representative fibre cross sections are shown in Supplementary Fig. 2 and Video 3. In transmission POM, the birefringence texture loses uniformity with increasing fibre thickness, see Supplementary Fig. 2a–f and Video 3. All slices are significantly brighter when aligned with their belt plane at $\pm 45°$ to the polarisers (d–f) than for parallel orientation (a–c), but the contrast is reduced the thicker the sample. This shows that the control of helix orientation is reduced the thicker the fibre, explaining why the colour saturation under ambient illumination is not as strong in thicker fibres, like that studied in Fig. 4 of the main paper. We speculate that thicker fibres require stronger anisotropic deswelling to ensure a uniformly vertical helix orientation, but with the current solution this can only be achieved by adding more DCM solvent. However, this would make the precursor liquid too fluid, leading to break-up due to the Plateau–Rayleigh instability. A better strategy may be to change the mandrel material to a porous or swellable one, allowing solvent extraction also downwards to boost the anisotropic deswelling, as was successfully employed for large flat films.[3] This requires a significant redesign of the fibre production equipment, in particular concerning production of the mandrel in a material such as polydimethylsiloxane. We are in the process of investigating this strategy and will report the results elsewhere.

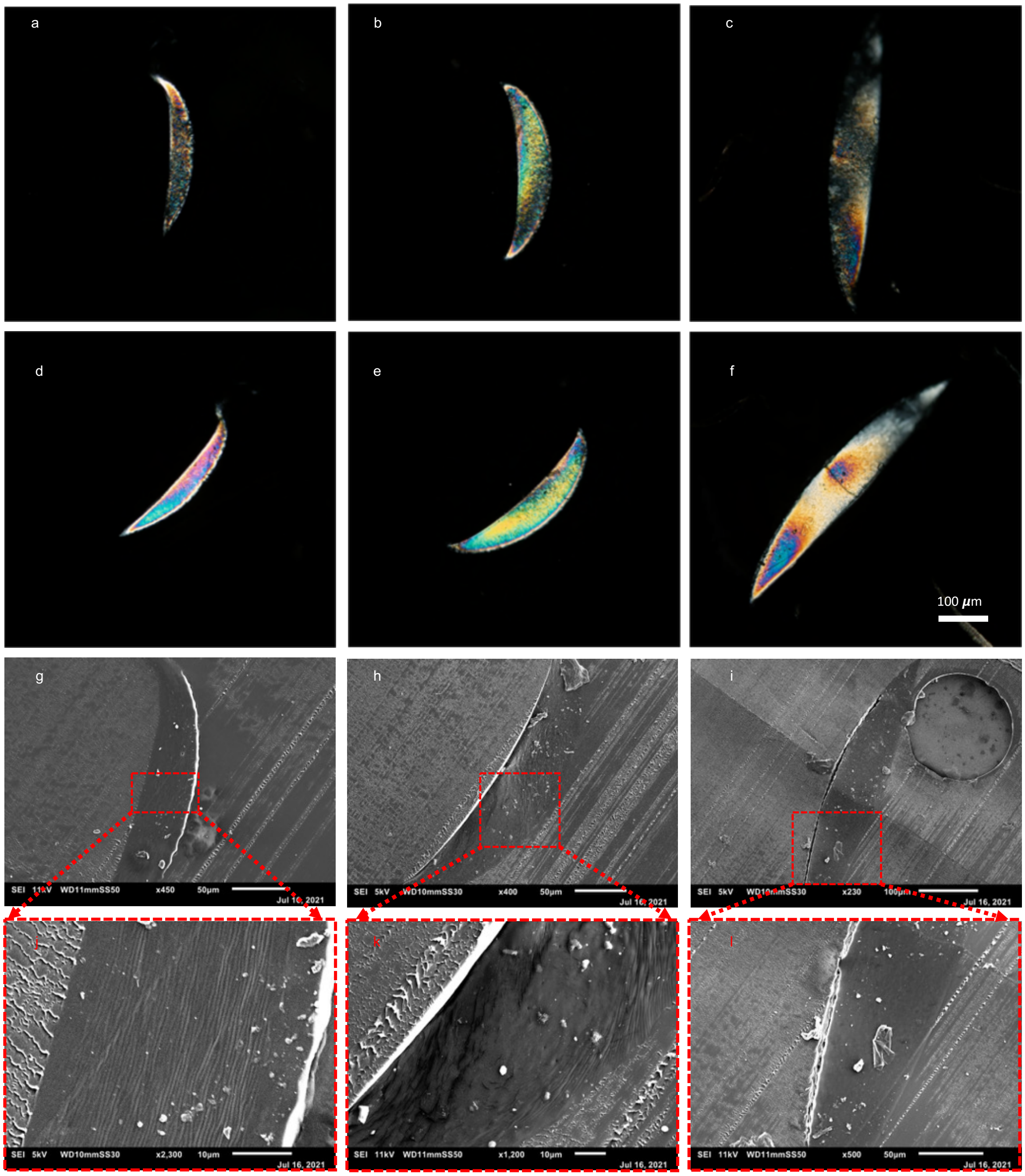

Supplementary Fig. 2. Crossed-polariser microscopy and SEM images of 5 μm thick cross sections from CLCE fibres with different thickness. The sections were obtained as explained in Supplementary Fig. 1 by embedding each CLCE fibre (made from LCO1) into NOA glue, curing the glue, and then slicing transverse to the fibre using a microtome with 5 μm step. The columns show slices from three fibres, increasing in thickness from left to right. The two top rows show POM images (extracted from Supplementary Video 3) and the two bottom rows show SEM images. In the top row, each slice is oriented for maximum extinction while in the second row it is oriented for maximum brightness. The reduction with increasing fibre thickness in contrast between the two orientations indicates reduced helix alignment quality. In the SEM images, evidence of uniform helix alignment is seen, as lines running perpendicular to the helix, only in the zoomed-in image of the thinnest fibre (j)
.

Supplementary Note 2: Microscopic characterisation of fibre under oblique illumination

A fibre of the initially red-retroreflecting type studied in the main paper is investigated microscopically for different angles of illumination in Supplementary Fig. 3. As predicted by Eq. (1) in the main paper, the reflection colour is blue-shifted upon inclined illumination, changing the appearance in the microscope from red to greenish-yellow, with variations along the curved edges. Overall, the colour is similar regardless of whether the light is inclined along or perpendicular to the fibre, as expected for an average helix orientation perpendicular to the fibre belt plane.

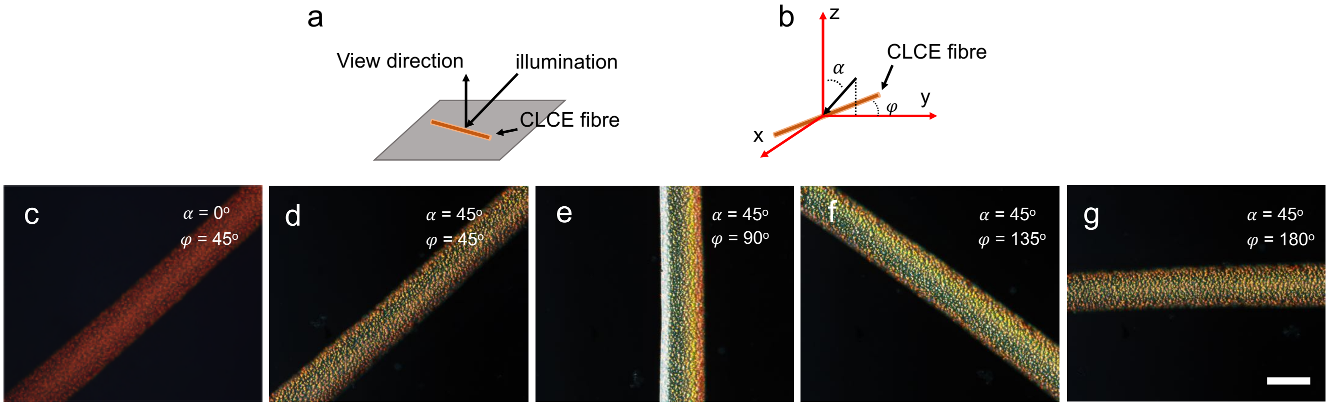

Supplementary Fig. 3. Dependence of reflection colour on illumination angle. a, Schematic showing the illumination and observation directions with respect to the fibre and **b**, definitions of the polar angle α with respect to the substrate plane and the azimuthal angle φ for defining the illumination direction. **(c–g)**, Reflection POM appearance of a fibre with λ_0 in the red regime in the relaxed state, as a function of varying α and φ. (Scale bar in **g** is 200 μm)

Supplementary Note 3: Mechanochromic response of fibres made from different LCOs

Because the fibres made from LCO2 and LCO3 have smaller mechanical response range than those made from LCO1 (see Extended Data Fig. 5), we conduct detailed spectroscopic measurements on the LCO1-derived fibres (main paper Fig. 3 and Extended Data Fig. 7). A confirmation of qualitatively similar mechanochromic response of fibres made from LCO2 and LCO3 is provided in Supplementary Video 9 and in Supplementary Fig. 4a, the latter with still frames extracted from the former.

Both fibres have ground state orange retroreflection colour, as shown in the POM images in Supplementary Fig. 4b–c. Under ambient light, the diffuse illumination gives both fibres an unsaturated pink-orange tone, but upon elongation the colour saturates, turning clearly green at around $\varepsilon_{xx} \approx 0.45$ and blue at $\varepsilon_{xx} \approx 0.85$. When relaxing the strain, the colour returns immediately, while it takes a few seconds for the fibres to retract to their original lengths.

Supplementary Note 4: Mechanochromic response of fibres made with different ground state reflection colour

Since the ground state λ_0 can be tuned by the amount of chiral dopant, we can easily make fibres that start out with different reflection colour, and because our eyes are more sensitive to a small wavelength change near the center of the visible spectrum (green-yellow) than towards the edges (red and violet, respectively), we can make fibres that give their most vivid change in perceived colour at different strain levels. To demonstrate the concept we produce three fibres based on LCO1 with different concentrations of chiral dopant, giving them λ_0 in the relaxed state that lies in the green, orange and red, respectively, and then subject them simultaneously to increasing strain levels. Supplementary Video 10 shows the experiment under ambient light as seen by a standard camera and as carried out at the POM, the latter also shown in Supplementary Fig. 5. Under ambient illumination, the relaxed fibres appear blue, green and orange; this is due to the multiple illumination directions in this setting, giving rise to blue-shifted apparent colour. In the following, we discuss the microscopic experiment carried out with collimated illumination along the viewing direction.

The fibres are shown in the order that the chiral dopant concentration decreases from left to right, i.e., the ground state colour changes from green to orange to red in this direction. The left-most fibre has its belt plane somewhat twisted when relaxed, hence the reflection colour initially appears only along one side, but the entire fibre becomes colourful during the experiment as the elongational strain untwists the belt plane. Also the right-most fibre shows reflections mainly along one side, but here it is most likely due to variations in the initial helix orientation. Again the reflection colour intensity increases upon strain, here because the compression perpendicular to the belt plane upon uniaxial elongation along the fibre aligns the helix vertically,[7] in the same way as anisotropic deswelling aligns the helix in the uncrosslinked precursor during solvent evaporation.

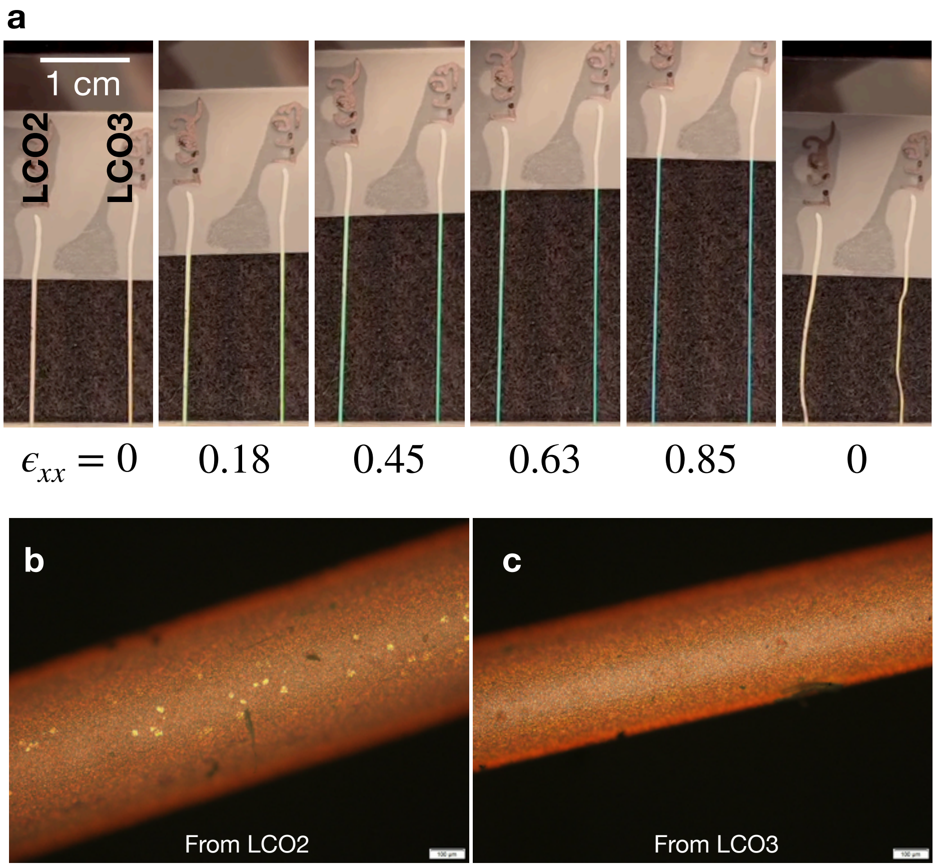

Supplementary Fig. 4. Mechanochromic colour shifts of CLCE fibres made from LCO2 and LCO3. a, Two fibres made from LCO2 (left) and LCO3 (right) are subjected to increasing tensile strain ε_{xx} while observing the reflection colour under ambient illumination. **b–c,** Reflection POM photography with collimated illumination of the LCO2-derived fibre (b) and the LCO3-derived fibre (c) reveal that both have ground state λ_0 corresponding to orange retroreflection.

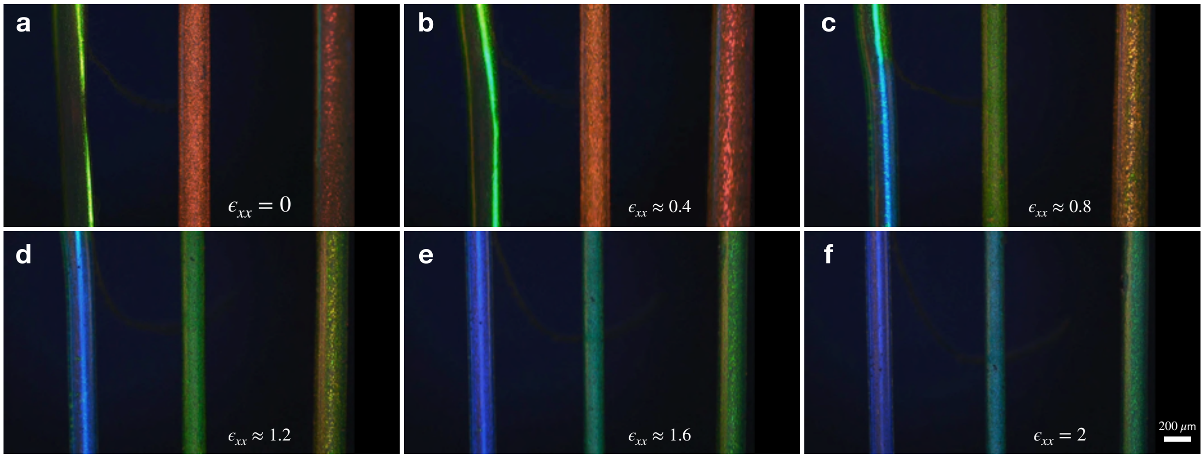

Supplementary Fig. 5. Mechanochromic colour shifts of CLCE fibres with different ground state colour. Reflection POM images of three fibres made from LCO1 but with varying amount of chiral dopant, giving them ground state λ_0 in the green, orange and red, respectively, subjected to identical and simultaneous elongational strain up to three times extension.

As the fibres are stretched together, the initially green fibre gives its strongest colorimetric fibre change, as experienced by the human eye, for low strains, changing from green to cyan at $\varepsilon_{xx} \approx 0.5$ and then to deep blue at $\varepsilon_{xx} = 0.9$. The initially orange fibre makes its most apparent colour transition at slightly greater strain, first showing almost no difference and then turning clearly green at $\varepsilon_{xx} = 0.8$. Meanwhile, the right-most fibre, with λ_0 initially in the red, makes its switch to green only at about $\varepsilon_{xx} = 1.2$. While λ_0 of course changes continuously for all three fibres, the colour change as experienced by human viewers can thus be tuned to different strain ranges.

This variation in colour response of the three fibres is quantified in Supplementary Fig. 6a, which shows λ_0 as obtained by spectrophotometer investigation of each fibre as a function of strain. Because of the different starting colours, the green fibre has a shorter response range than the others, since the reflection wavelength exits the visible spectrum at much lower strains. The full reflection spectra without polariser are shown for the initially green fibre in Supplementary Fig. 6b, showing qualitatively very similar behavior to the initial red fibre in Fig. 3 in the main paper but with all spectra blue-shifted as a result of the higher chiral dopant concentration.

The reflection spectra of the initially red-retroreflecting fibre (a different fibre from that in Fig. 3 in the main paper) are recorded through right- and left-handed circular polarisers, respectively, the results shown in Supplementary Fig. 6c–d. As expected for a CLCE,[6] the reflection is initially circularly polarised with the same handedness as the helix (here right-handed),

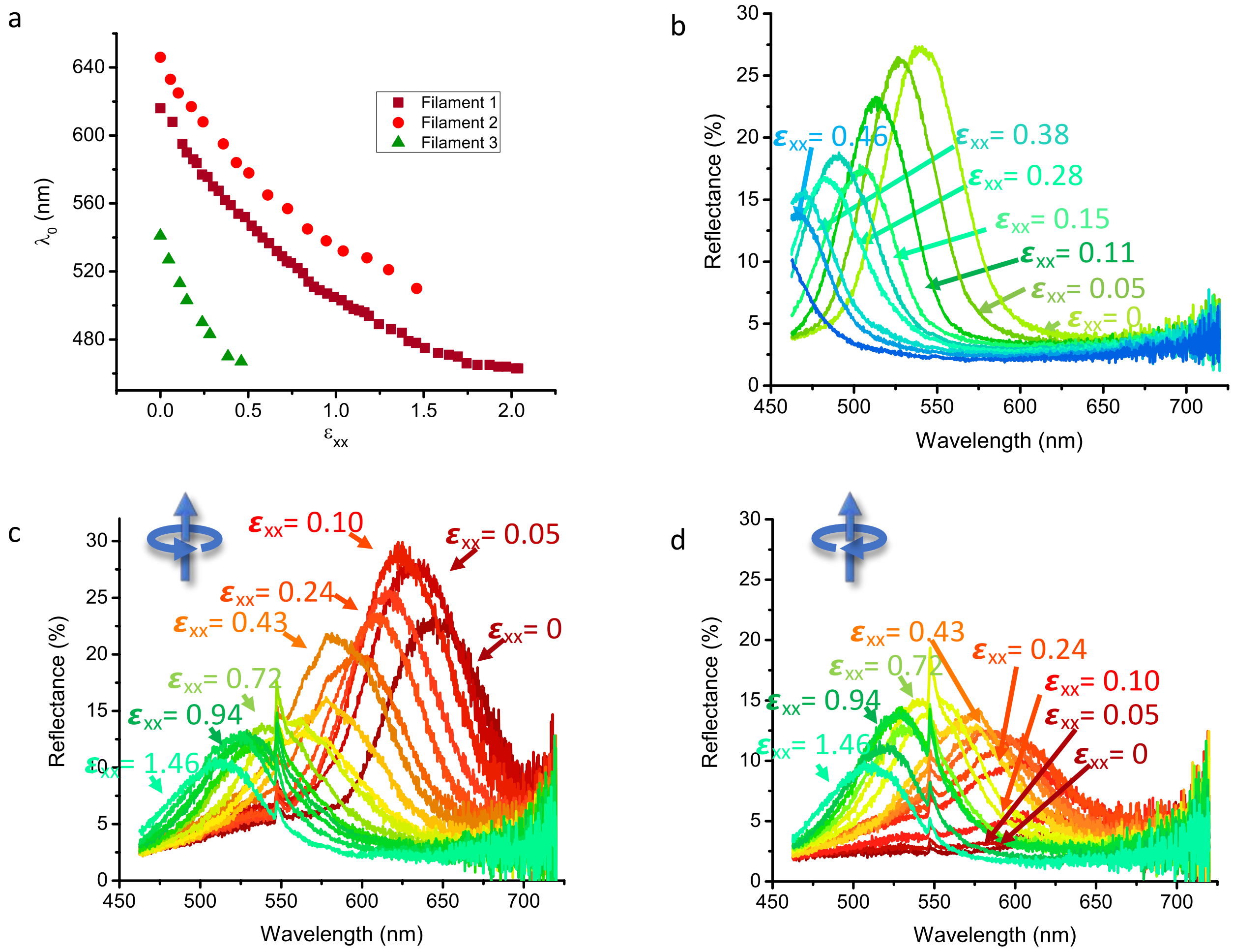

Supplementary Fig. 6. Quantitative mechanochromic response of CLCE fibres with green, orange and red ground state colour, respectively. a, The $\lambda_0(\varepsilon_{xx})$ behavior for three fibres made from LCO1 but with varying amount of chiral dopant, giving them ground state retroreflection colours that are green, orange and red, respectively. **b,** The reflection spectra of an initially green reflecting CLCE fibre under stepwise increasing strain, as measured without polariser. **c–d,** The reflection spectra of an initially red reflecting CLCE fibre under stepwise increasing strain, as measured through a right- (c) and a left-handed (d) polariser, respectively.

but with increasing strain the ellipticity changes and also the left-handed channel contains the selective reflection. In the range $\varepsilon_{xx} \approx 0.7 \rightarrow 1$, the left-handed channel even shows a slightly stronger peak than the right-handed, whereas the peak area is nearly identical in both channels for $\varepsilon_{xx} \approx 1.5$.

Supplementary Note 5: Testing machine washing and long-term durability of CLCE fibres

To confirm the excellent durability of the CLCE fibres also when they are sewn into garments for practical use, we launder the fabric shown in Fig. 4e–h in the main paper in a conventional consumer grade washing machine (LG) at 40°C, using one Ariel All-in-1 pod as detergent, one Calgon tab against calcium-rich water, and one dose of Soupline softener. A standard cotton washing program was run, ending with 400 rpm centrifugation. While the cloth loses some saturation in its black colour, no detectable effect can be seen on the CLCE fibre, neither in its relaxed state nor in its mechanochromic response, as shown in Supplementary Fig. 7.

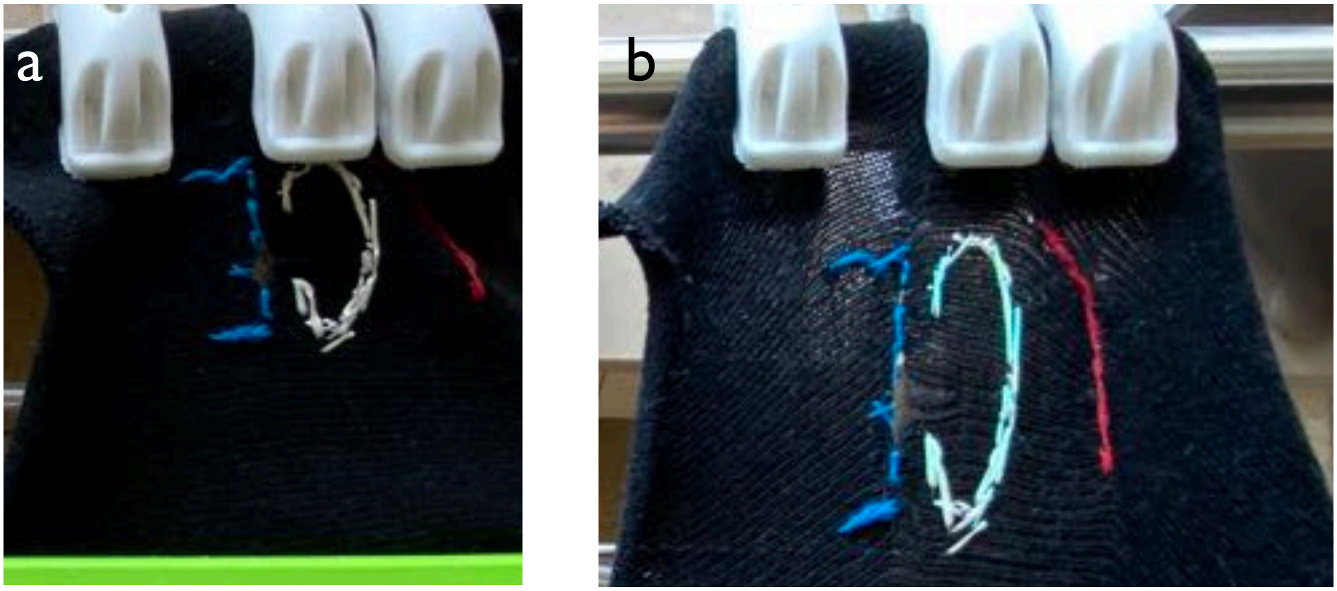

Supplementary Fig. 7. Laundry resistance of CLCE fibres. The cloth with a CLCE fibre sewn into it in the shape of a 'C', after a regular 40° laundry with 400 rpm centrifugation (it is still wet). The cloth is photographed with a regular mobile phone camera under daylight illumination through a window, unstrained (**a**) and uniaxially strained (**b**).

After the above experiment, the fabric was carried by one of the authors in a hand bag for more than 6 months, where it was subject to significant wear and contact with numerous every-day items and materials. The fabric was also taken out for testing the mechanochromic response at irregular intervals. As shown in Supplementary Fig. 8, the black cloth looks worn down after this long-term experiment, the black colour being less saturated. In contrast, the CLCE fibre shows no apparent change in visual appearance, displaying just as good mechanochromic response upon straining of the cloth. Significantly, it also has not picked up any debris or dust, although the fibre had no protective coating of magnesium stearate or similar.

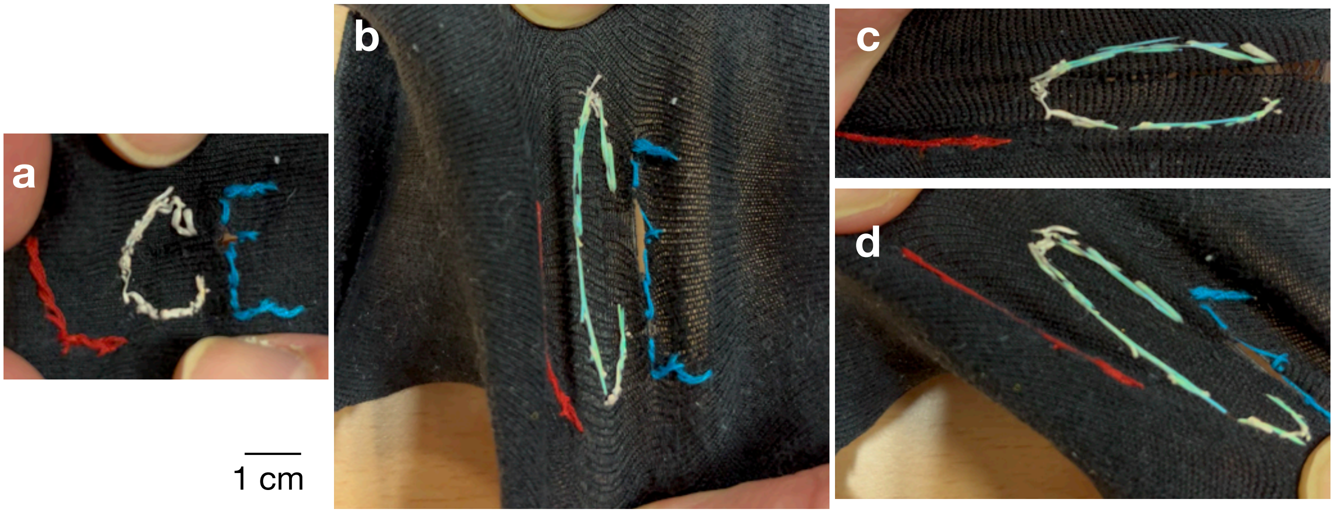

Supplementary Fig. 8. Long-term durability of CLCE fibre. The above cloth after six months of carrying in a handbag after the laundry experiment, in the relaxed state (a) and subject to tensile strain in different directions (b–d). The cloth is photographed with a regular mobile phone camera under standard office light illumination.

Supplementary discussion

Origin of striations in SEM images of fibre cross section

The parallel lines oriented along the belt plane seen in high-magnification SEM of the section of the thinnest fibre (Supplementary Fig. 2j and Fig. 1k in the main paper) may be expected to run perpendicular to the helix, thus indicating that the helix is generally well aligned along the belt plane normal. However, their periodicity is on the order of 1.5 μm, which is much greater than the $p \approx 400$ nm expected from Eq. (1) in the main paper for the red selective reflection that we observe in POM from this fibre in its pristine state. Actually, there is no reason for different orientations of the director to exhibit different contrast for electron imaging, and one can thus not expect that the helical modulation should, on its own, be detectable by SEM. We note that we only see the lines clearly in the thinnest slice, although all slices clearly have helical director modulation as witnessed by their selective reflection behaviour confirmed during optical characterisation. This shows that strong confinement is key for the lines to appear in SEM, and that they are not a direct 'fingerprint' of the cholesteric helix.

To understand their origin, we must consider an aspect of CLCEs—indeed of any LCEs—that we do not focus on in this paper, namely that they actuate if the degree of orientational order is reduced, for instance by heating, compressing along **n** and expanding in the perpendicular directions[1]. During SEM investigation, in particular at higher magnifications, the heating required for actuation may result from the electron beam exposure. If we observe a CLCE perpendicular to its helix during actuation, a periodic set of protrusions and retractions may arise from the surface, as seen by Feng et al.[2]. Since the helix is perpendicular to **n**, CLCEs try to expand in the direction of the helix axis, but in our case this is prevented by the cured NOA glue surrounding which is not actuating in response to the heating. The resulting frustration may cause undulating instabilities, especially for tightly confined thin fibres, investigated with high magnification SEM that gives rise to strong heating. We believe this is the reason for the appearance of the lines with a rather large periodicity.

Formation of cholesteric structure during relaxation of CLCE precursor filament

In the interval between photos during the early part of the experiment shown in Extended Data Fig. 2, we temporarily rotate the sample stage to confirm that the filaments turn dark when they are oriented along either polariser. With a first-order λ plate inserted, the total birefringence increases when the λ plate optic axis is along the filament and it decreases when it is perpendicular. These observations reveal that the filaments exhibit long-range order throughout the process, although they are drawn from LCO-DCM mixtures that are isotropic at rest. The birefringence is initially uniaxial positive with optic axis along the filament axis. This can be explained by a non-equilibrium flow-induced and flow-aligned paranematic state, as illustrated in a highly idealised schematic way in Fig. 1f in the main paper, as can be expected from the production process.

With time, the flow-aligned state is gradually replaced by the thermodynamically stable cholesteric phase with its helical director configuration. With anisotropic deswelling aligning the helix vertically, this should reduce the effective fibre birefringence in transmission POM over time, which is also seen in some fibres but not in all in the upper row of Extended Data Fig. 2. Some fibres remain bright, indicating imperfect helix orientation or remaining flow-induced non-helical arrangement. In fact, these filaments generally show stronger birefringence than the final crosslinked CLCE fibres, suggesting that the hand-drawing and deposition on a glass slide is not fully representative of the filament drawing process on the rotating mandrel. However, the lower row of the figure shows that also the filaments on the glass slide gradually become red in reflection microscopy between crossed polarisers. As verified by the colour being unchanged upon sample stage rotation, this is due to selective reflection, showing that the cholesteric phase is developing with sufficient fraction having vertical helix orientation to give the filaments their uniformly red colour. This experiment does not rule out that the helix may also develop along other directions, and the remaining birefringence in parts of the fibres in the upper row shows that, indeed, such developments are likely. However, the many experiments carried out on the final crosslinked fibres, described in Supplementary Note 1 and in the main paper, give a totality of evidence that the helix predominantly aligns perpendicular to the fibre belt plane in the final state, even if this alignment is imperfect particularly in thick fibres.

Colour variation between centre and edges of fibres

Both in Extended Data Fig. 3 and in Fig. 3b–j in the main paper, we note that the fibre edges display a slightly different reflection behavior compared to the central regions; in general, the edges show red-shifted colour compared to the centre. We hypothesise that this is due to a lower degree of anisotropic deswelling at the thinnest points of the fibre, which dry too quickly for anisotropic deswelling to compress the helix as much as it does in the center.[4] This would mean that the edges have longer initial λ_0 than the centre. For the fibre with red λ_0 in Fig. 2 and 3a in the main paper, the red-shifted reflection at the edges ends up in the infrared, explaining why it is not prominent in the ground state fibre. Only after some initial strain has been applied, the reflection colour along the edges shifts to red and then orange, following a mechanochromic response that appears delayed compared to the central regions, which show visible selective reflection from the start.

Mechanical properties of CLCE fibres with different degree of crosslinking

Quantitatively, the CLCE fibres are about twice as soft in the linear regime as the commercial rubber band, and the latter has much greater breaking strain and stress. The trend shown for the three CLCE fibre types highlights the impact of crosslinking,

and we may thus conjecture that oligomer redesign tailored for increased crosslink density, for instance by choosing monomers with lateral reactive substituents to allow more than two crosslinks per oligomer, will significantly expand the mechanical operating range of the CLCE fibres, getting closer to that of the regular rubber band. Since the most crosslinked CLCE fibre in this study showed excellent mechanochromic response, following the theoretical prediction throughout and somewhat beyond the initial linear regime (compare Extended Data Figures 4 and 5), we believe this will hold also for more crosslinked CLCE fibres. The initial modulus may be tuned by shifting the balance between mesogens and chain extender in the oligomer, a hardening to be expected for shorter or less flexible chain extenders.

Impact of dye doping

While the amount of Sudan Black that we add in this study does not apparently alter the reflection colour of our fibre, beyond the desired reduction in non-selective scattering, we note that the addition of dye may have an interesting impact on the Bragg reflection. This is because the presence of a dye within the CLCE structure may affect the average refractive index $\bar{n}$, which affects the retroreflection colour as $\lambda_0 = \bar{n}p$ (see equation (1) in the main paper). Given that dyes have a complex refractive index, the imaginary component of which represents the absorption (across the visible spectrum for black dyes), the exact impact of the dye and the dependence of its concentration are non-trivial to assess. We have started an in-depth study of these effects and will report the outcome elsewhere.

Impact of CLCE fibre stickiness

We have done several experiments similar to the hand sewing experiment in Fig. 4i in the main paper using slightly shorter CLCE fibres without any PVA coating, as the stickiness is only a problem for fibres that are so long that they tend to coil on themselves during sewing, leading to knots and loops. Commercial elastomeric fibres are routinely coated with release agents like magnesium stearate to reduce stickiness and thus facilitate handling. Given the chemical similarity we expect such procedures to work very well also for our CLCE fibres.

Supplementary References

1. Zhang, P., Zhou, G., de Haan, L. T. & Schenning, A. P. 4d chiral photonic actuators with switchable hyper-reflectivity. *Adv. Funct. Mater.* **31**, 2007887 (2021).
2. Feng, W., Chu, L., de Rooij, M. B., Liu, D. & Broer, D. J. Photoswitching between water-tolerant adhesion and swift release by inverting liquid crystal fingerprint topography. *Adv. Sci.* **8**, 2004051 (2021).
3. Kizhakidathazhath, R. *et al.* Facile anisotropic deswelling method for realizing large-area cholesteric liquid crystal elastomers with uniform structural color and broad-range mechanochromic response. *Adv. Funct. Mater.* **30**, 1909537 (2020).
4. Guenthner, A. J. *et al.* Dynamics of Hollow Nanofiber Formation During Solidification Subjected to Solvent Evaporation. *Macromol. Theory Simul.* **15**, 87–93 (2006).
5. Warner, M., Terentjev, E. M., Meyer, R. B. & Mao, Y. Untwisting of a cholesteric elastomer by a mechanical field. *Phys. Rev. Lett.* **85**, 2320–2323 (2000).
6. Cicuta, P., Tajbakhsh, A. & Terentjev, E. Photonic gaps in cholesteric elastomers under deformation. *Phys. Rev. E* **70**, 011703 (2004).
7. Frka-Petesic, B., Kamita, G., Guidetti, G. & Vignolini, S. Angular optical response of cellulose nanocrystal films explained by the distortion of the arrested suspension upon drying. *Phys. Rev. Mater.* .
